# A Model-Based Approach to Support Validation of Medical Cyber-Physical Systems

**DOI:** 10.3390/s151127625

**Published:** 2015-10-30

**Authors:** Lenardo C. Silva, Hyggo O. Almeida, Angelo Perkusich, Mirko Perkusich

**Affiliations:** Embedded Systems and Pervasive Computing Laboratory, Federal University of Campina Grande, 882 Aprígio Veloso Street, Universitário, Campina Grande 58429-900, Brazil; E-Mails: hyggo@dsc.ufcg.edu.br (H.O.A.); perkusic@dee.ufcg.edu.br (A.P.); mirko.perkusich@embedded.ufcg.edu.br (M.P.)

**Keywords:** medical cyber-physical systems, early validation, simulation, formal methods, model-based development, clinical scenarios, medical devices, patient model

## Abstract

Medical Cyber-Physical Systems (MCPS) are context-aware, life-critical systems with patient safety as the main concern, demanding rigorous processes for validation to guarantee user requirement compliance and specification-oriented correctness. In this article, we propose a model-based approach for early validation of MCPS, focusing on promoting reusability and productivity. It enables system developers to build MCPS formal models based on a library of patient and medical device models, and simulate the MCPS to identify undesirable behaviors at design time. Our approach has been applied to three different clinical scenarios to evaluate its reusability potential for different contexts. We have also validated our approach through an empirical evaluation with developers to assess productivity and reusability. Finally, our models have been formally verified considering functional and safety requirements and model coverage.

## 1. Introduction

Cyber-Physical Systems (CPS) have been pointed to as a powerful solution for improving healthcare services [1,2]. According to Lee *et al.* [3], the traditional clinical scenarios are closed-loop systems in which the caregivers are the *controllers*, medical devices act as *sensors* and *actuators*, and patients are the physical *plants*. As a distinct class of CPS, Medical Cyber-Physical Systems (MCPS) modify this scenario by introducing additional computational entities to help the caregiver to control the *plant*, *i.e.*, in the decision support [4]. MCPS are context-aware, life-critical systems with patient safety as the main concern, demanding rigorous processes for validation to guarantee user requirement compliance and specification-oriented correctness.

For different reasons, MCPS validation is inherently more complex than traditional systems validation [3]. From the *physical* perspective, MCPS developers must consider patient uncertainty and heterogeneity. Due to insufficient understanding of human body dynamics in response to treatments, it is not possible to consider one deterministic patient behavior. Also, each patient is unique, with a different behavior according to his age, clinical history and environmental aspects [5,6,7]. On the other hand, system validation with real patients without jeopardizing the patient’s health is a complex problem [3,8,9], what makes early validation a promising approach. In the development process, early validation occurs at design time, allowing analyzing critical aspects of a system before implementation. Model-based simulation is commonly applied for early validation [10].

From the *cyber* perspective, MCPS developers must deal with device heterogeneity, dependability, and reliability. US Food and Drug Administration (FDA) classified approximately 1700 different generic types of devices, not including the specializations. FDA registered an increase in the number of recalls caused by software failures in medical devices [11]. These failures can be related to software design, implementation and testing procedures, but are also due to the growing complexity of the medical device usage environment, with increased connectivity and interoperability.

Putting together the *physical* and *cyber* perspectives also brings new challenges. How to consider different patient and device behaviors to build a single and integrated MCPS solution? How to validate each aspect of the MCPS behavior for different types of patients and devices? How to reuse existing patient and device data for simulating different MCPS, for different clinical scenarios? How to integrate new knowledge about patients and devices to improve quality and productivity in future MCPS validation? In this work, we focus on facing these challenges.

Several related works have been proposed within MCPS area. For example, Integrated Clinical Environment (ICE) is a conceptual functional model and standard that establishes the requirements for the safe integration of medical devices and other equipment in a medical system [12]. Based on this conceptual model, researchers construct models that represent medical systems to assist design and increase the error resistance. An ICE can be considered as a high-level framework for MCPS description. Based on ICE, Pajic *et al.* [13] present a clinical scenario for patient-controlled analgesia, which can benefit from the closed-loop approach to control drug delivery.

Jiang *et al.* [14,15] published a series of works to establish a procedure to ensure the correct operation of cardiac pacemaker devices using the concept of a closed-loop system. In Jiang *et al.* [14] the authors provide an environment for closed-loop testing, in which the patient model, specifically a formal model of the human heart, is the control center of a cardiac pacemaker system. The goal is to evaluate the device’s operational safety and effectiveness based on the patient’s condition.

Miller *et al.* [16] describe an approach based on digital mockups as a method for MCPS testing. In this case, the models are used to test medical devices by providing input and analyzing the outputs to enable the evaluation of their functionalities. The application scenario of the approach involved a mechanical ventilation device connected to an artificial lung model.

There are also works related to patient modeling. For instance, Van Heusden *et al.* [17] propose an artificial pancreas model for patients with type 1 mellitus diabetes based on control theory. Their goal is to improve glucose control. One of its advantages is that it incorporates the patient’s medication (insulin) reaction. Other approaches use models of clinical contexts to validate and verify the control of software. Kang *et al.* [18], King *et al.* [19], Simalatsar and De Micheli [20] and Li *et al.* [21] focused on MCPS design issues. They coupled their solutions to specific formalisms and associated tools. Murugesan *et al.* [22] considered compositional verification of a medical system at multiple levels of abstraction, with different formalisms used at each level. Their contribution is towards a general, scalable and practical approach to layered verification of properties in complex cyber-physical systems.

In general, such approaches present the following limitations to face the challenges mentioned before:

1. the models for clinical scenarios are restricted to the specific purpose of the system, without support to adapt the respective patient and device models for a new clinical context of interest, making its reuse unfeasible;
2. the physical process simulation ignores important aspects of the clinical scenario dynamics, including external disturbances (e.g., user interventions) and particular reaction of each patient to the same stimuli (e.g., drug administration);
3. the patient models either focus on variables of interest for specific clinical scenarios or neglect the relationship between the four human vital signs: heart and respiratory rates, blood pressure, and body temperature. The absence of this aspect contradicts the actual behavior of human beings [5,6]. Furthermore, when these models are formally defined in mathematical language they are not represented computationally and *vice-versa*. Therefore, these models have limited applicability to other scenarios.

In this work we propose a formal approach for early validation of MCPS using simulation models based on *Actor-Oriented Design* [23]. We provide a reusable model library composed of two types of models: patients and devices. Patient models are based on medical guidelines and clinical databases. Device models are built from the technical specification of sensor devices and actuators. The models are built as reusable components, being customizable enough to be reused in different MCPS, and can also be adapted or extended to fit unpredicted MCPS design requirements.

We propose a composition process to support developers on putting the pieces together to build their specific MCPS models, using existing models for patients and devices. The integrated MCPS models can be simulated and analyzed to identify undesirable behaviors at design time. Extension and adaptation guidelines for each type of model is also provided, allowing creating new reusable models to enrich the model library. This complete process to improve reuse and productivity in the early validation of MCPS is the main contribution of this work.

Our approach has been applied to three different clinical scenarios to evaluate its reusability potential for different contexts. Also, we have successfully validated our approach though an empirical evaluation with developers, applying the Goal-Question-Metric method [24]. Finally, the simulation models have been successfully verified using Simulink Design Verifier [25]. We formally verified functional and safety requirements and model coverage of one of the three clinical scenarios presented.

The remainder of this article is organized as follows. Section 2 describes our model library, giving details on how to build new models for patients and devices. In Section 3 we detail the composition process to create MCPS models based on existing models for patients and devices. Section 4 discusses the validation of our approach, including the application for three different scenarios and empirical evaluation. In Section 5 we present the formal verification of the models. Finally, Section 6 presents conclusions and future works.

## 2. Model Library: Building Reusable Models

The model library is a set of reusable patient and device models aiming to increase the potential for reuse and productivity during the MCPS early validation. We have defined guidelines to allow MCPS developers to adapt existing models or build their own models according to the system requirements, enriching the library along the time.

In this work, the modeling process is based on the *Actor-Oriented Design* (AOD) paradigm [23], a design methodology based on components called actors. This methodology represents a formal model of concurrency, in which an actor is a computational agent that has an independent thread of control and communicates through asynchronous message exchange. To construct the models, we used the *Ptolemy II* modeling tool [26], which is an extensible AOD-based software framework that supports experimentation. Its emphasis is in concurrent components, using well-defined computation models that govern the interactions among these components.

In this section, we detail how to build patient and medical device models for composing the model library. By applying the guidelines, one can develop models for devices and patients to be used in different contexts. The same guidelines have been used to build the existing models, which are also described in this section.

### 2.1. Building Patient Models

Patient models are formal models built based on medical guidelines and clinical databases. A patient model consists of a set of attributes/parameters (e.g., demographics) that defines the patient profile, and a set of components to generate synthetic data related to vital signs and physiological parameters. In addition, a set of mathematical models is incorporated to represent the dynamics of key biological, pathological, and pharmacological processes in the body of a patient undergoing drug treatment, as a response to different stimuli. In general, these mathematical models are defined by the *Pharmacology*. According to Katzung [27], *Pharmacology* is the science that studies substances that interact with living systems through chemical processes, activating or inhibiting normal body processes. Interactions between a drug and the human body are divided into two classes: (i) the actions of the drug on the body are named *Pharmacodynamics (PD) processes*; and (ii) the actions of the body on the drug are named *Pharmacokinetics (PK) processes*. In other words, the *PK processes* govern the absorption, distribution, and elimination of drugs, while the *PD processes* are concerned with the chemical actions on the organism.

A patient model can represent the partial or total behavior of the human body, including specific organs or completely organic systems, respectively. Such representations may incorporate simple aspects, such as a set of vital signs and physiological parameters, and complex aspects, such as the response to the administration of a specific drug. Modeling patient behavior always raises a question about how to deal with the non-deterministic nature of the patient. According to Lee [4], MCPS naturally combine models in such a way that determinism is not preserved. However, he demonstrates that deterministic MCPS models have practical feasibility. The degree to which the model imitates the thing being modeled (or vice versa) defines its value, however, it is always approximate. For example, Li *et al.* [9] highlight that there are no good models to describe the complex behavior of patients, usually described by ODEs, and that these models may not fit into existing hybrid systems model checking tools. On the other hand, in a short finite time horizon, techniques such as linear regression and linearization can describe patients’ basic and complex behaviors, respectively. In this work, we approach this problem by outlining comprehensive processes to describe basic behaviors of patients (e.g., vital signs) using multivariate regression models (e.g., generalized linear models) and complex behaviors (e.g., insulin and glucose kinetics) using control theory approaches (e.g., state-space models and controller design).

The procedure to build a patient model consists of four stages detailed in the following subsections. The explanation for each step is given using an example of a Diabetic Patient Model representing the behavior of diabetic patients.

#### 2.1.1. Stage 1—Choosing a Clinical Database

For this initial stage, we choose a dataset of patients with personal data and vital signs records and physiological parameters. This clinical dataset must have at least 30 patient records to apply multiple regression analysis, which is the minimum amount required to achieve a given level of accuracy and confidence for the regression models. The sample size is important because it can affect the statistical power of the regression model and in result generalization [28]. If the dataset contains a large volume of data, it is necessary to characterize a population of interest. This process consists of applying a set of rules to the real patient data obtained from the clinical database. The aim is to reduce the larger volume of records contained in the selected dataset.

For example, to build the Diabetic Patient Model we selected the dataset *MIMIC II Clinical Database v2.6* [29], whose access was authorized by *PhysioNet.org*. This dataset contains clinical data from Intensive Care Unit (ICU) patients. Due to the large amount of data records at this clinical dataset, we applied rules to characterize the population of interest.

**Figure 1:**
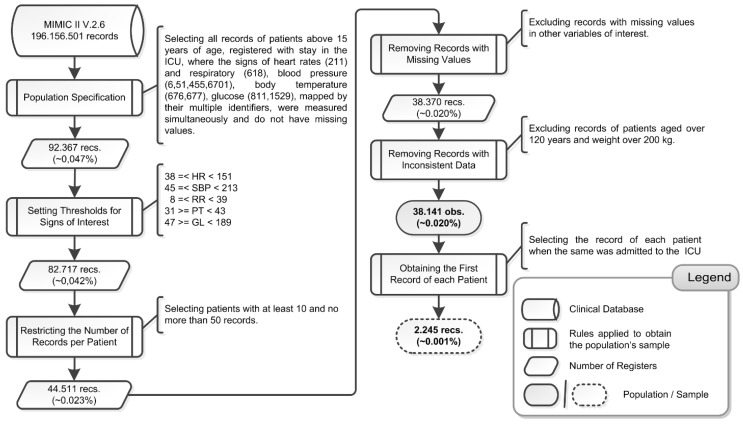
Process to Characterize the Population of Interest.

The population contained 38,141 observations from 2245 patients, in which approximately 37.4% were female and 62.6% male. From this population, we obtained a sample with one observation for each patient at the moment they were admitted in the ICU. We present a summary of the process to characterize the population of interest and sampling procedure in Figure 1, whose sample size represents only approximately 0.001% of the total amount of records from the clinical database.

The next step is to select the set of variables to perform the statistical analysis. Since the vital signs and physiological parameters provided by the patient model are based on thresholds, we established the parameters that defined these thresholds in accordance with *Clinical Guidelines*. We use these clinical guidelines to help identifying the probable regression models predictor variables for each vital sign considered in the patient model.

For example, to select the predictor variables for the statistical analysis for the Diabetic Patient Model, we used the clinical guidelines described in [30,31,32,33,34,35]. We selected eight probable predictor variables: *gender*, *weight*, and *height* as demographic variables; heart rate (*hr*), respiratory rate (*rr*), systolic blood pressure (*sbp*), and body temperature (*pt*), as vital signs; and blood glucose level (*gl*), as a physiological parameter. Table 1 presents descriptive statistics about the dataset extracted from this process.

**Table 1:** Summary Measures of the Population of Interest.

Variable	Mean	Std. Dev.	CV	Minimum	Maximum
*hr_value*	87.315	14.263	0.163	40.00	150.00
*sbp_value*	116.518	20.049	0.172	48.00	212.00
*rr_value*	19.216	5.628	0.293	8.00	38.00
*pt_value*	37.251	0.739	0.020	31.70	41.44
*gl_value*	118.761	27.802	0.234	47.00	188.00
*weight*	84.300	20.730	0.246	33.00	200.00
*height*	169.804	10.379	0.061	124.50	231.10

Std. Dev.: Standard Deviation; CV: Coefficient of Variation.

#### 2.1.2. Stage 2—Obtaining a Statistical Model

With the dataset and the variables selected for analysis, the goal at this stage is to obtain a regression model for each variable of interest. Descriptive statistics should be analyzed to choose the appropriate regression technique. In this sense, we compare the summary measures of the sample obtained with the population to check the representativeness of the study variables. In addition, we analyze the distribution of data from boxplots and histograms, and the linear correlations among the selected variables. Thus, we identify the data profile contained in the sample. Based on the data profile, we use Multivariate Regression Models (MRM) to generate the vital signs. These statistical regression models are integrated to provide the patient model basic dynamics.

**Figure 2:**
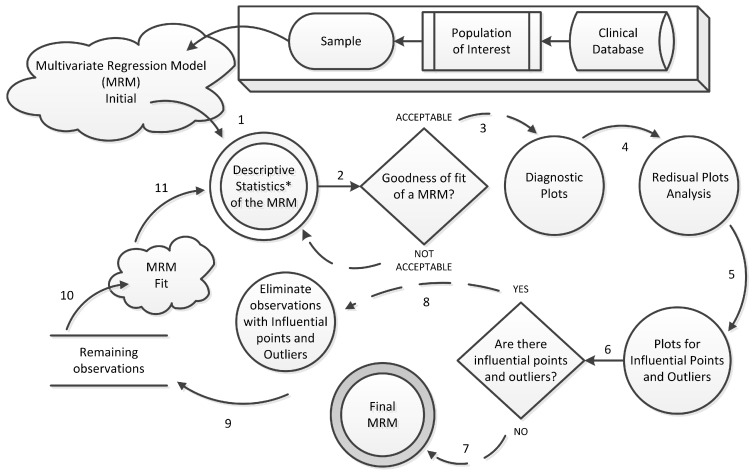
The Fit Process of the Multivariate Regression Model for Each Vital Sign of Interest.

The workflow to fit the MRM for each vital sign is shown in Figure 2. Given a sample extracted from the population of interest, we obtain an initial multivariate regression model. Therefore, we analyze the descriptive statistics of this regression model including a set of predictor variables. If the model statistics do not provide sufficient evidence about the model quality (*i.e.*, model predictions are still NOT ACCEPTABLE), it is necessary to modify the initial regression model. For instance, it may be necessary to include other more significant variables or remove less significant ones. An example of quality metric used to assess the fit of a regression model is the determination coefficient $R^{2}$. On the other hand, if the regression model has good quality indicators (*i.e.*, model predictions are ACCEPTABLE), it is necessary to generate a set of diagnostic plots that allow a more detailed analysis of the model. Examples of diagnostic plots are the envelope plot (*Normal Q-Q Plot*), leverage points, influential points, and residuals *versus* fitted values plot.

Envelope plots are useful to check the regression models fit. Leverage points may interfere in adjusted values close to them and regression coefficients estimate. Influential points may interfere in the model parameters estimated values. The residuals versus fitted values plot is useful to assess the assumptions of the regression model (e.g., any visible trends would show a dependence of errors on the predictor variable) [36]. After analyzing the diagnostic plots of each regression model, the developer should decide which of them would be used to generate the vital signs that they represent. This means that the synthetic data generated by the patient model must be compatible with the sample used in the statistical analysis. The iterative fit process shown in Figure 2 must be applied to all regression models to be included in the patient model.

For the example of Diabetic Patient Model, we used the Generalized Linear Models (GLM), a class of MRM, to obtain the regression models. In the GLM, we assume that the response variable follows the exponential family distribution and the predicted values are calculated from a link function [37]. Hence, we fit a regression model for each vital sign considered (*i.e.*, *hr*, *rr*, *sbp*, and *pt*). For example, considering the respiratory rate variable (*rr*), we used a *Normal Inverse Model* in the form of Equation (1) with canonical link function defined in Equation (2) to predict the respiratory rate sign (*GLM_RR*).

(1) $$\hat{\mu }={\hat{\eta }}^{-\frac{1}{2}}$$

where in this case $\hat{\mu }$ is the mean frequency of respiratory rate, and

(2) $$\hat{\eta }={\hat{\beta }}_{0}+\sum_{\begin{array}{c} i=1 \end{array}}^{4}{\hat{\beta }}_{i}X_{i}+\sum_{\begin{array}{c} j=2 \end{array}}^{4}{\hat{\beta }}_{5j}X_{5j}+\sum_{\begin{array}{c} k=6 \end{array}}^{7}{\hat{\beta }}_{k}X_{k}$$

$\hat{\eta }$ is the systematic component, $\beta_{0}$ correspond to the intercept and $\beta_{1-7}$ to the coefficients of the variables *hr*, *sbp*, *pt*, *gl* and *group*, as well as the interactions between *hr×sbp* and *hr×gl*, respectively. The *group* variable is used according to the patient classification, given the values of the other predictor variables. Therefore, the $X_{5j}$ variable assumes the value *1* according to the *j* value that specifies the patient group, and the value *0* for all other possible *j* values.

For the remaining variables (*hr*, *sbp*, and *pt*) we used *Gamma Linear Regression Model* given by Equation (3), whose variance function is more restrained than in the *Normal Inverse Model*:

(3) $$\hat{\mu }={\hat{\eta }}^{-1}$$

where $\hat{\mu }$ is the mean frequency of vital signs that we want to model; in this case *hr*, *sbp* and *pt*. Since the procedure to map regression models for all vital signs are similar, these regression models were omitted.

#### 2.1.3. Stage 3—Developing an AOD Model

At this stage, the regression models of each vital sign are mapped to a formal model based on Actor-Oriented Design. For this purpose, it is necessary to choose a set of parameters to define the patient profile. The patient model consists of the integration of the regression models to generate the inherent synthetic data of the vital signs of interest, together with demographic attributes that define the patient profile.

Regression models mapped to an AOD model must allow interaction among them and provide the behavioral dynamics to the patient model. Thus, the developer can change the value of a specific vital sign during simulation, and the values of other vital signs will be automatically modified according to their respective regression models.

In Table 2, we describe how to map regression model elements to AOD elements, including an explanation about each AOD element. All AOD elements are available as graphical widgets in the Ptolemy II user interface.

**Table 2:** Mapping from Regression Model to AOD Paradigm.

Regression Model’s Term	AOD Actor	Actor’s Explanation
Intercept	Constant	Produce a constant output. The value of the output is that of the token contained by the value parameter, which by default is an integer with value 1.
Coefficient	Scale	Multiplies the input by a constant given as a parameter.
Predictor variable	Input port	An IOPort that defines the data type of an input.
Interaction between predictor variables	Expression	On each firing, evaluates an expression that may include references to the inputs, current time, and a count of the firing. The ports are referenced by the identifiers that have the same name as the port.
Math Symbols	AddSubtract	A polymorphic adder/subtractor. This adder has two input ports, both of which are multiports, and one output port, which is not. Data arriving on the input port named plus will be added, and data arriving on the input port named minus will be subtracted.
$\hat{\eta }$	Expression	-
$\hat{\mu }$	Output port	An IOPort that defines the data type of an output.

**Figure 3:**
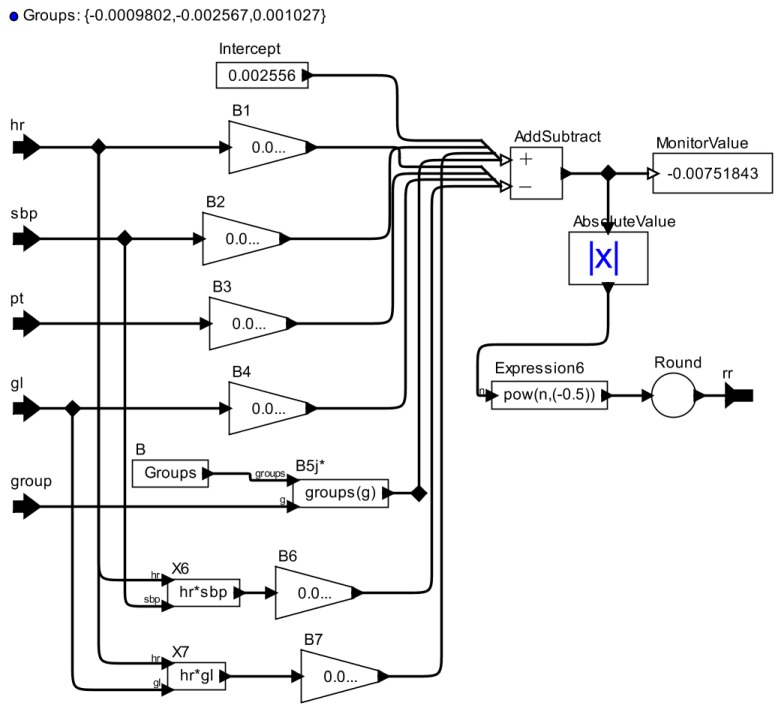
*GLM_RR* for the Patient Model.

For example, to map the regression model for *rr* given by Equation (4) to the AOD model illustrated in Figure 3, we represent the intercept by a *Constant* actor; coefficients by a *Scale* actor; predictor variables by *Input* ports; and interactions between variables (e.g., $X_{6}$ and $X_{7}$) by *Expression* actors. The *group* predictor variable, which identifies the group to which the patient belongs based on his vital signs, is represented by a *Constant* actor. This actor maps the vector of coefficients that can be used in the equation according to the input *group* through an input port, as explained in Equation (2). To represent the signs before each term of (4), such as $-0.0001206X_{3}$, we link each term output to the respective input of *AddSubtract* actor, which defines each signal (*i.e.*, plus or minus). In the case of *GLM_RR*, it is necessary that the absolute output value of *AddSubtract* actor is obtained using an *AbsoluteValue* actor to calculate the predicted value of the vital sign *rr*, as defined by Equation (1). To represent this equation in AOD, we also used an *Expression* actor. The output of the AOD model representing the regression model for a vital sign is rounded to the closest integer value using a *Round* actor. The result is the synthetic data generated for the modeled vital sign, in this case, respiratory frequency sign.

(4) $$\begin{array}{c} \hat{\eta }=0.002556-0.00005756X_{1}+0.00005381X_{2}-0.0001206X_{3}-0.00001715X_{4}-\\ 0.0009802X_{52}-0.002567X_{53}+0.001027X_{54}-\\ 0.0000004994X_{6}+0.0000001579X_{7} \end{array}$$

**Figure 4:**
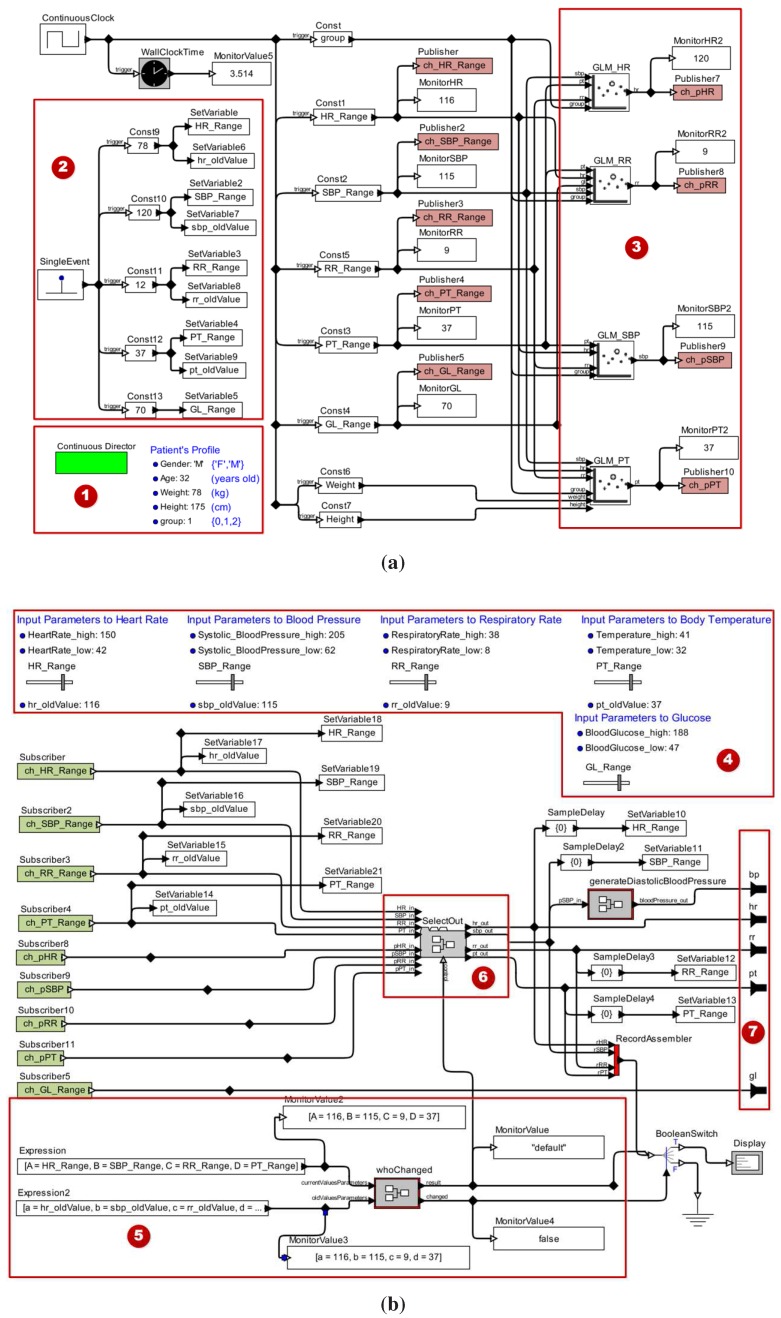
Example of a Patient Model. (**a**) Regression Models to Estimate Vital Signs Data; (**b**) Submodel to Provide Information to the Medical Device Models.

The same mapping process can be used for the other vital signs. In Figure 4, we illustrate a patient model considering all vital signs, with patient profile and regression models already integrated.

In Figure 4a, we present a patient model incorporating the patient characteristics and the regression models of the four vital signs. We have highlighted the following key elements: (1) the patient model configuration parameters, including the Continuous Director, which determines their execution semantics; (2) the substructure of the model that specifies the vital signs initial values and physiological parameters represented in the model; (3) the elements labeled by the *GLM_<vital_sign>* pattern that denotes the regression models for each vital sign modeled. The model parameters are the basis for generating the values for the vital signs provided by the patient model. Moreover, the specifications of the thresholds of each vital sign allows the developer to manipulate them during the simulation to represent different health conditions for the patient model. Consequently, the behavior analysis of the MCPS may be carried out for various situations.

The second part of a patient model is illustrated in Figure 4b. The highlighted elements are: (4) the parameters that define thresholds for each signal to assure that these signals remain within the range of values considered in the construction of the regression models; (5) the logic used to identify which vital sign was changed by the user at a given time during the model simulation; (6) the component developed to select the model’s correct output according to user intervention; and (7) ports that provide the patient model communication interfaces with the medical devices models for data acquisition.

Notice that our current patient model has only output ports: vital signs (*i.e.*, *hr*, *rr*, *sbp*, and *pt*) and physiological parameters (*i.e.*, *gl*). To receive feedback control actions from actuator models, input ports must be added to the patient model through the *Ptolemy II* framework. Additionally, the patient model must be adapted to represent the pharmacokinetic model dynamic behavior corresponding to the drug type to be administered. In other words, we need a patient model that not only simulates the basic condition of the health characterized by vital signs of an individual, but also clinically responds to the treatment. In this case, the actuator models are used to simulate the drug administration.

In general, the pharmacokinetics and pharmacodynamics processes for a specific drug are mathematically represented by one or more ordinary differential equations (ODE), typically characterizing non-linear models. An ODE is mapped to the AOD paradigm by a *LinearStateSpace* or *ContinuousTransferFunction* actor, whose the mapping process will be explained later in our example. Despite the human body to be non-deterministic, it works as a control system, in which each control signal must achieve a desired behavior represented by a reference signal [38]. The response to the treatment of a patient to a drug can be mathematically represented by a PID (proportional-integral-derivative) controller that acts to lead the controlled signal to its reference signal. For a continuous-time parallel PID controller [39], the transfer function is described in Equation (5).

(5) $$G\left(s\right)=K_{p}+K_{i}\left(\frac{1}{s}\right)+K_{d}\left(\frac{N\cdot s}{s+N}\right)$$

where $K_{p}$ is the gain of the *proportional* term, $K_{i}$ is the gain of the *integral* term, $K_{d}$ is the gain of the *derivative* term, and the filter coefficient *N* sets the location of the pole in the derivative filter. In our example, the *proportional* term represents the insulin infusion rate, which is directly proportional to the *error*. In our work, the error is the difference between the *current* glucose level (controlled signal) and *desired* glucose level (reference signal). The derivative term depends on the rate of change in the glucose level of the patient. The higher the rate of increase of glucose level, the higher the rate of increase in the insulin dosage administered to the patient. Lastly, the *integral* term evaluates the insulin infusion rate by means of the integral of the *error*, forcing the error signal to zero in each iteration.

To design a PID controller, a well-known practice is to linearize the ODEs. This facilitates the tuning process of the PID controller at a given operating point for the controlled signal. For example, the Matlab^®^ [40] provides the *Control System Toolbox*, a software package that contains a set of functions defined for Control Systems Engineering. The Ptolemy II has also the PID actor to map the equation (5) to AOD paradigm.

To demonstrate this stage from our example, we modeled the effect of insulin to control a patient glucose level. According to Bergman, Phillips and Cobelli [41], the system regulating the glucose concentration is envisioned as being divided into two parts: (*a*) the glucose-dependent segment, which determines the plasma insulin; and (*b*) the insulin-dependent segment, which determines the plasma glucose. In the glucose-dependent segment (*i.e.*, *Glucose Kinetic*) occurs the pancreas and insulin-degrading tissues. In the insulin-dependent segment (*i.e.*, *Insulin Kinetic*) occurs glucose producing and utilization tissues. The authors proposed two “minimal” mathematical models to describe the dynamic glucose and insulin responses during an intravenous glucose tolerance test. Using these models, characteristic parameters of insulin sensitivity and pancreatic responsivity are generated. This set of mathematical models has been named *Bergman’s Minimal Model* (BMM) [41].

The BMM assumes a closed-loop relationship between a minimum number of three compartments: G(t) is the instantaneous glucose concentration in blood (mg/dL); X(t) is the effective amount of insulin used in disappearing plasma glucose (min${}^{-1}$); and I(t) is the instantaneous insulin concentration in plasma (*μ*/mL). Each compartment is described by one ODE as shown in Equations (6)–(8).

(6) $$\frac{dG(t)}{dt}=-p_{1}\left[G\left(t\right)-G_{b}\right]-X\left(t\right)G\left(t\right)+\left[D\left(t\right)+C\left(t\right)\right]$$

(7) $$\frac{dX(t)}{dt}=-p_{2}X\left(t\right)+p_{3}\left[I\left(t\right)-I_{b}\right]$$

(8) $$\frac{dI(t)}{dt}=-n\left[I\left(t\right)-I_{b}\right]+\gamma {\left[G\left(t\right)-h\right]}^{+}t+r\left(t\right)$$

where D(t) and C(t) are parameters that represent disturbances to the patient. For instance, the rate of glucose absorbed after food intake and the rate of glucagon infusion to avoid hypoglycemia, respectively. $G_{b}$ and $I_{b}$ are the *Basal* levels of glucose and insulin in blood.

The remaining parameters of the nonlinear BMM are: the coefficient $p_{1}$, which defines the effectiveness of insulin when glucose remains at $I_{b}$; and $-p_{2}/p_{3}$, the effect of insulin on glucose level to maintain it within the normal range of an individual, equivalent to the sensitivity insulin; *n* is the rate at which insulin is being used up in plasma ($min^{-1}$); *h* is the lowest value of blood glucose above which endogenous insulin is secreted (mg/dL); *γ* is the rate of endogenous release of insulin when glucose is infused exogenously and concentration of blood glucose is above *h* threshold ($\left[\mu \;U/mL\;min^{-2}{\left(mg/dL\right)}^{-1}\right]$).

In this work, we use a linearized form of *Bergman’s Minimal Model* presented by Khan *et al.* [42] that is described by Equations (9)–(11).

(9) $$\frac{dG(t)}{dt}=-p_{1}\left[G\left(t\right)-G_{b}\right]-\bar{X}\cdot G\left(t\right)-\bar{G}\cdot X\left(t\right)+\bar{G}\cdot \bar{X}+\left[D(t)+C(t)\right]$$

(10) $$\frac{dX(t)}{dt}=-p_{2}X\left(t\right)+p_{3}\left[I\left(t\right)-I_{b}\right]$$

(11) $$\frac{dI(t)}{dt}=-n\left[I\left(t\right)-I_{b}\right]+\tau \cdot r\left(t\right)$$

where $\bar{G}$ and $\bar{X}$ are the average values of G(t) and X(t). The controller’s output r(t) (μU/mL/min) acts to maintain blood glucose level equal to $G_{b}$, and *τ* is a unit conversion factor of r(t) to U/h.

The *Linearization* process results in a linear model which approximates a nonlinear system specifically for an operating point. According to Franklin, Powell and Emami-Naeini [43], it is possible to obtain a linearized model that is valid near an equilibrium point (*i.e.*, operating point). Whenever the system state differs from the operating point, both the linearization process and the controller design must be performed again.

Thus, let the state vector x(t) and inputs vector u(t), where $x\left(t\right)={\left[\begin{array}{ccc} G(t) & X(t) & I(t) \end{array}\right]}^{T}$ and $u\left(t\right)={\left[\begin{array}{ccc} D(t) & C(t) & r(t) \end{array}\right]}^{T}$. The nonlinear BMM can be defined by $\dot{x}=f\left(x,u\right)={\left[\begin{array}{ccc} f_{0}\left(x,u\right) & f_{1}\left(x,u\right) & f_{2}\left(x,u\right) \end{array}\right]}^{T}$ that represents the equations (1-3). We determine a linearized model around the operating point $\left(x_{0},u_{0}\right)$, in which $x_{0}={\left[\begin{array}{ccc} G_{0} & X_{0} & I_{0} \end{array}\right]}^{T}$ and $u_{0}={\left[\begin{array}{ccc} D_{0} & C_{0} & r_{0} \end{array}\right]}^{T}$, such that Equation (12) implies Equation (13).

(12) $${\left.\dot{x}\right|}_{\begin{array}{c} x=x_{0}\\ u=u_{0} \end{array}}=0\Rightarrow \left[\begin{array}{c} f_{0}\left(x_{0},u_{0}\right)\\ f_{1}\left(x_{0},u_{0}\right)\\ f_{2}\left(x_{0},u_{0}\right) \end{array}\right]=\left[\begin{array}{c} 0\\ 0\\ 0 \end{array}\right]\Rightarrow$$

(13) $$\Rightarrow \left[\begin{array}{c} -p_{1}\left(G_{0}-G_{b}\right)-X_{0}G_{0}+D_{0}+C_{0}\\ -p_{2}X_{0}+p_{3}\left(I_{0}-I_{b}\right)\\ -n\left(I_{0}-I_{b}\right)+\tau \cdot r_{0} \end{array}\right]=\left[\begin{array}{c} 0\\ 0\\ 0 \end{array}\right]\Rightarrow \left\{\begin{array}{c} G_{0}=\frac{p_{1}G_{b}+D_{0}+C_{0}}{p_{1}+\frac{p_{3}\tau \cdot r_{0}}{p2\cdot n}}\\ X_{0}=\frac{p_{3}\tau \cdot r_{0}}{p_{2}\cdot n}\\ I_{0}=I_{b}+\frac{\tau \cdot r_{0}}{n} \end{array}\right.$$

By applying the linearization process demonstrated by Ogata [44], the linearized BMM may be defined as $\dot{x}-f\left(x_{0},u_{0}\right)=A\delta x+B\delta u$, as shown in Equation (14) and represented by Equation (15).

(14) $$\Delta \dot{x}=\underset{{\left.\frac{\partial f}{\partial x}\right|}_{\begin{array}{c} x=x_{0}\\ u=u_{0} \end{array}}}{\underset{︸}{A}}\Delta x+\underset{{\left.\frac{\partial f}{\partial u}\right|}_{\begin{array}{c} x=x_{0}\\ u=u_{0} \end{array}}}{\underset{︸}{b}}\Delta u$$

(15) $$\begin{array}{c} \left[\begin{array}{c} \dot{G(t)}\\ \dot{X(t)}\\ \dot{I(t)} \end{array}\right]=\left[\begin{array}{ccc} -p_{1}-X_{0} & -G_{0} & 0\\ 0 & -p_{2} & p_{3}\\ 0 & 0 & -n \end{array}\right]\left[\begin{array}{c} \Delta G(t)\\ \Delta X(t)\\ \Delta I(t) \end{array}\right]+\\ \;\left[\begin{array}{c} 1\\ 0\\ 0 \end{array}\right]\Delta D(t)+\left[\begin{array}{c} 1\\ 0\\ 0 \end{array}\right]\Delta C(t)+\left[\begin{array}{c} 0\\ 0\\ \tau \end{array}\right]\Delta r(t) \end{array}$$

Considering $\dot{x}-f\left(x_{0},u_{0}\right)=A+\delta x+B\delta u$, then we write Equation (15) as Equations (9)–(11) given by Equation (16).

The behavior of the patient model H(s) proposed by Khan *et al.* [42] is based on the *Laplace Transform* of the Equations (9)–(11) given by Equation (16),

(16) $$H\left(s\right)=T_{1}\left(s\right)\cdot R\left(s\right)+T_{2}\left(s\right)\left[D(s)+C(s)\right]+T_{3}\left(s\right)\cdot I\left(s\right)$$

where R(s) is the disturbance response, I(s) is the *Laplace transform* of impulse and $T_{n}\left(s\right)$ are the transfer functions, in which n∈{1,2,3}. The transfer functions relate the inputs for the patient model. Khan *et al.* [42] present more information regarding the usage of *Bergman’s Minimal Model*.

**Figure 5:**
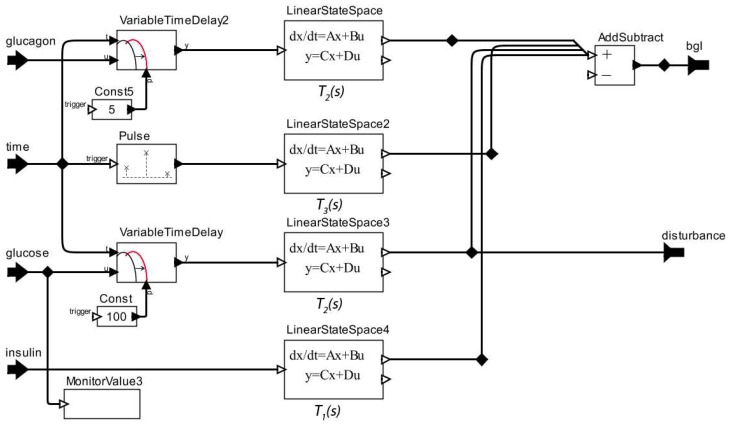
Model of the Effect of Insulin to Control a Patient Glucose Level.

Since we understand the dynamics to be included in the patient model, we represent such dynamics using Ptolemy II as shown in Figure 5.

Because a linear dynamic system may be represented by different forms, such as differential equations, transfer functions, state-space, and pole-zero, it is possible to transform the system representation from any one form to another [43]. Matlab allows us to perform the conversion automatically by using specific commands. In this work, we transformed the patient model proposed by Khan *et al.* [42], from *transfer functions* to an equivalent representation in *state-space* using the command tf2ss. The analytical relationship between the state-space description and the transfer-function description is demonstrated by Franklin, Powell and Emami-Naeini [43]. We used the state-space representation because the state of the patient model related to glucose requires an *initial state*. This initial value for a specific state cannot be defined by means of a transfer function.

**Figure 6:**
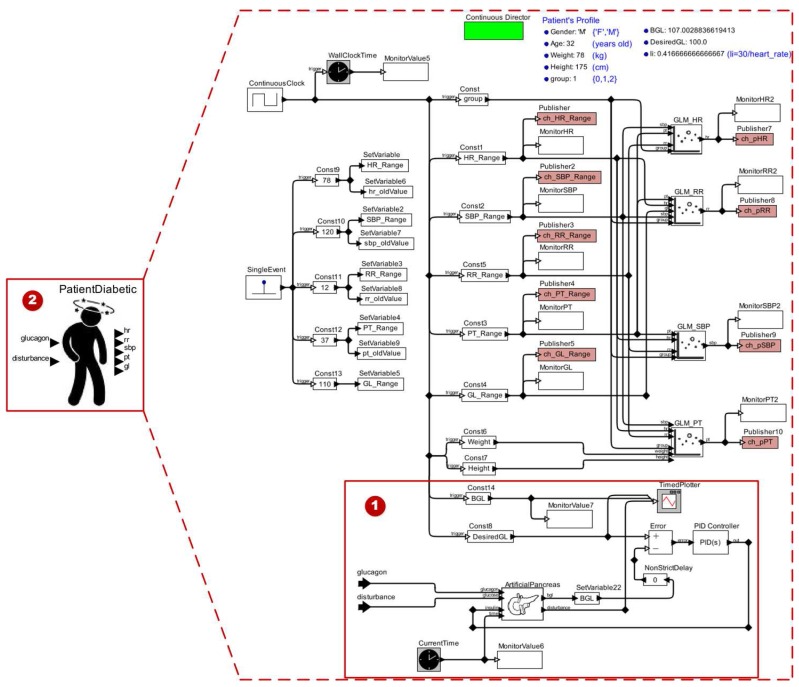
Diabetic Patient Model.

The next step of this stage is to transform the model shown in the Figure 5 to an actor model and include it in our original patient model. Then we must add input ports to the patient model to receive the actions of the actuator devices, as well as of disturbances. Afterwards, we need to establish the relationships among Diabetic Patient Model actors, aiming to represent the insulin response dynamic to the patient model. We show the result of this process in Figure 6, highlighting the key changes performed in the patient model: (1) the new actors and input ports added; and (2) the abstraction of the Diabetic Patient Model as an actor to be made available on the library of *Ptolemy II*.

#### 2.1.4. Stage 4—Validating the Patient Model

In this stage, the behavior of the patient model should be validated to assure its accuracy. This includes analyzing if the regression models correctly compute the generated data during the AOD model simulation. This stage is finished after evidencing the correct behavior of the patient model. Otherwise, the MCPS developer should perform adjustments.

**Figure 7:**
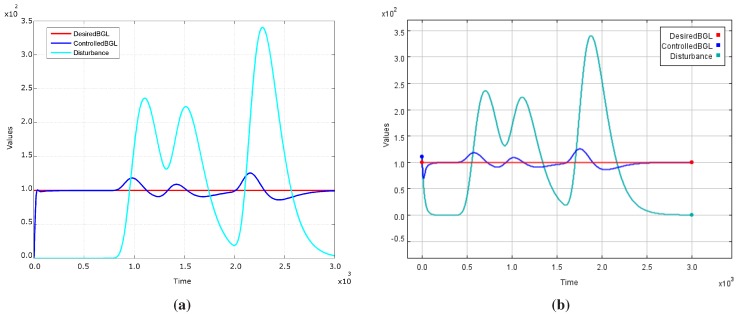
Comparative Result Between BGL Control System’s Original Model at *Simulink* and Replicated Model at *Ptolemy II* After Simulation of 3000 Time Units. (**a**) Simulink; (**b**) Ptolemy II.

To validate the Diabetic Patient Model, we replicated the blood glucose level (BGL) control system in *Ptolemy II* and compared the outputs after simulation as shown in Figure 7. We can observe that both BGL control system models have similar behavior. Thus, they present the same curve shape for blood glucose level (*ControlledBGL*) generated by the patient models. This also allows us to state that the patient models represented by both the *Simulink* and *Ptolemy II* respond similarly to disturbances. In other words, these patient models have equal embedded dynamics. On the other hand, the original patient model, differently from the changes we propose, does not consider an initial state for the glucose parameter. We can notice this behavior during the first one hundred time units of the simulation. Another observed change in Figure 7a,b are the offsets related to disturbance curve, which do not cause problems to validate the patient model behavior.

### 2.2. Building Medical Device Models

Medical device models are formal models built from the technical specification of sensor devices and actuators. Examples of such devices include blood pressure meters, scales, glucometers, and infusion pumps. Sensor models are capable of capturing health condition data of patients. Actuator models administer drugs to keep the patient model in stable condition. These drugs are administered to achieve a beneficial therapeutic effect on some process within the patient [27].

In general, sensor models have a simpler structure if compared to actuator models. This feature is related to the higher functional safety level required for these devices.

The process for building a medical device model consists of three stages. The explanation for each step is given in the following subsections, using an example of an insulin pump model.

#### 2.2.1. Stage 1—Choosing a Certified Medical Device

This stage aims to identify, analyze and select a technical specification that best describes the medical device features that developers want to model. The chosen medical devices must be certified by a regulatory agency such as the FDA [45].

For our example, we chose the user guide of a certified insulin pump system [46] to elicit the requirements to be implemented in this actuator model. We present in Table 3 the key features for proper functioning of the insulin pump. Since our goal is to simulate the medical device given its functional behavior, we ignore the device physical and electromagnetic characteristics.

**Table 3:** Technical Requirements Considered in the Modeling Process of the Insulin Pump.

Configuration Parameters	Functional Requirements	Safety Properties
1. Insulin Type: U100 ${}^{\left(1\right)}$	1. Operation Modes: Run/Stop;	1. Errors (E) / Alerts (A):
2. Cartridge Capacity: 3.15 mL ${}^{\left(2\right)}$	2. Delivery Modes: *Basal*, *Bolus* and *Corrective Bolus*;	a. E: “Cartridge Empty!”
3. *Basal* (U/h):	3. Checking the cartridge level;	b. A: “Cartridge Low Warning!”
Min. = 0.1	4. Programming standard *Bolus* dose;	c. A: “Cartridge Ok!”
Max. = 25.0	5. Administrating corrective *Bolus* dose;	2. State:
4. *Basal* Profile:	6. Providing information to the users.	a. “*STOP*”
a. Standard (# fixed dose/3min): 480		b. “*EXECUTING*”
b. Customized (# flexible dose/h): 24		
5. Standard *Bolus* (U): Max. = 25.0		
6. Administration Rate (dose/min)${}^{\left(3\right)}$:		
a. *Basal* Dose		
b. *Bolus* Dose		

${}^{\left(1\right)}$ Short-acting insulin or Rapid-acting insulin; ${}^{\left(2\right)}$ Equivalent to 315 units of insulin U100; ${}^{\left(3\right)}$ Simplification, since that in the specification the administration rate is 0.2 U/s.

Treatment of a diabetic patient by insulin therapy requires multiple insulin doses to be administered over the day. An endocrinologist prescribes at least two types of insulin dose to the diabetic patient; the *Basal* insulin and the *Bolus* type [47]. *Basal* insulin is used to keep blood glucose levels within the normal range of an individual during periods of fasting. By being long-acting, it is usually taken once or twice a day depending on the insulin. On the other hand, *Bolus* insulin is specifically taken at meal times to keep blood glucose levels under control after a meal. This is a rapid-acting insulin in which administration follows medical guidelines. This is a specific clinical scenario of insulin pump usage.

#### 2.2.2. Stage 2—Developing an AOD Model

**Figure 8:**
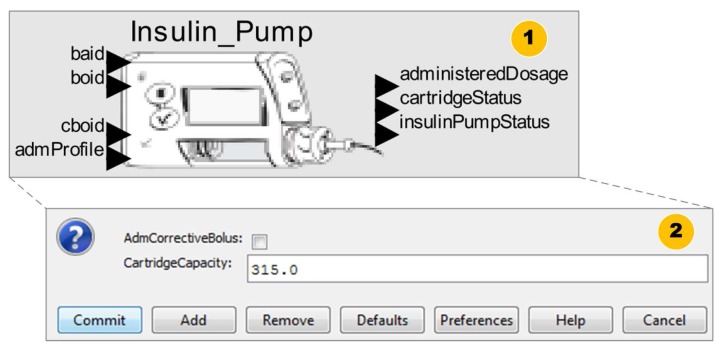
Insulin Pump Model.

At this stage, the medical device model is constructed according to the technical specification considering the key features of the medical device and safety properties for the proper behavior of the model. Once the requirements to model the insulin pump are defined, the next step is to construct the model. We present an overview of the insulin pump model in Figure 8.

We present the component *actor* used to abstract the functioning logic of the pump in Figure 8(1). This component instantiates the insulin pump model into a clinical scenario model. The insulin pump has as an input the insulin delivery profile identifier (*admProfile*), and a set of interfaces to receive the values corresponding to *Basal* insulin dose (*baid*), standard *Bolus* (*boid*), and corrective *Bolus* (*cboid*). Users can configure these parameters to suit their needs. This model provides as output a set of information about the pump’s state (*insulinPumpStatus*), the insulin level remaining in the cartridge (*cartridgeStatus*), and the amount of insulin administered during its execution.

As shown in Figure 8(2), the insulin pump model has other configuration parameters. For example, the cartridge capacity (*CartridgeCapacity*) coupled to the insulin pump and an option to immediately administer a corrective *Bolus* dose (AdmCorrectiveBolus) to the patient model. Users can configure these parameters during model simulation.

**Figure 9:**
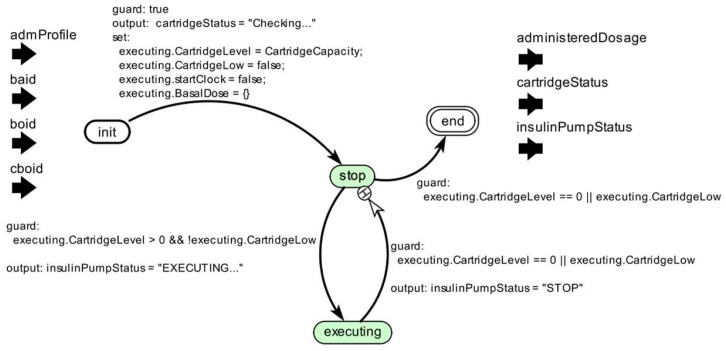
Insulin Pump Model’s Controller.

Furthermore, in Figure 9, we show the insulin pump’s control component, which is responsible to control the behavioral dynamics of the insulin pump during simulation.

To define it, we used the *Modal Model* concept, which consists of a finite set of behaviors and rules that govern transitions between them. The rules are captured by a *Finite State Machine* (FSM). Each behavior represents an operation mode for each state of the FSM. The dynamics of the FSM can be specified by multiple state’s refinements, in which each refinement defines the behavior of the system in that operation mode [48]. Silva *et al.* [49] present more details regarding this component.

We can generalize an FSM to admit continuous inputs and outputs and to combine discrete and continuous dynamics. This model of computation is known as an *Extended State Machine* (ESM) [48]. An ESM have a numerical state variable, in which the number of states depends on the number of distinct values that the variable can take. Therefore, to generate simulation cases for medical device models, we can combine structure-based control-flow-oriented and state/transition-oriented coverage criteria. Thus, it is possible that actuator models interact with the patient model, MCPS model, and other medical device models.

**Figure 10:**
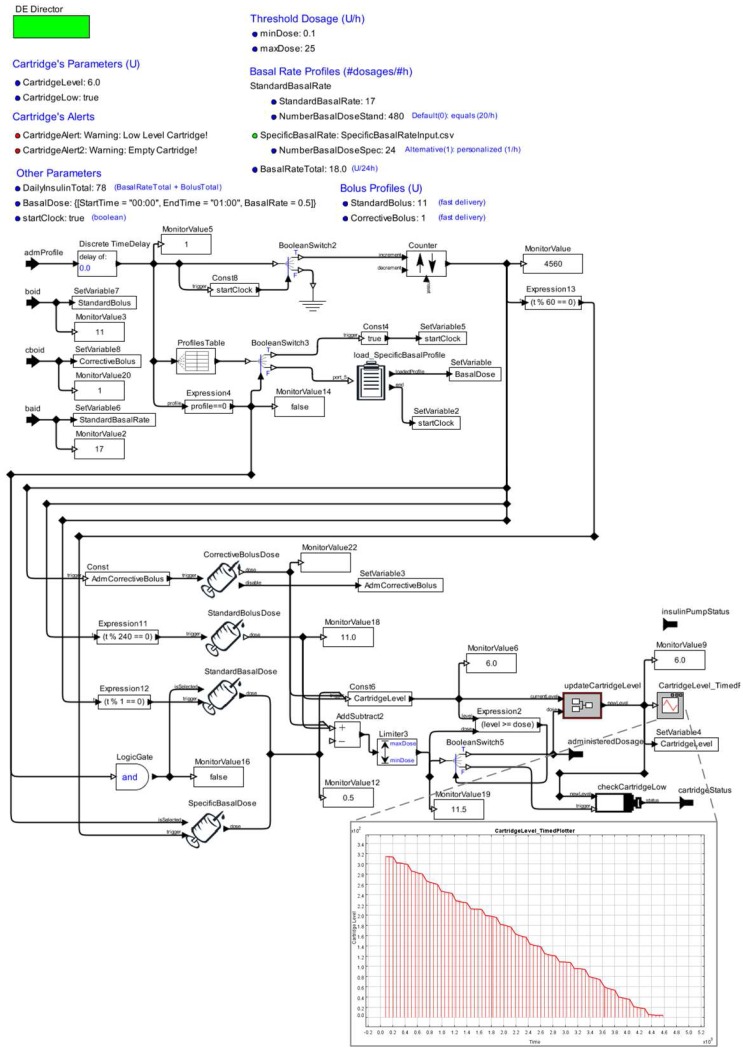
State’s Refinement of the Insulin Pump Model.

We show the state refinement model for the operation modes “*stop*” and “*executing*” in Figure 10. Such a refinement has a *Discrete-Event model*, in which interactions among actors occur at discrete points in time. Each interaction represents an event characterized by the exchange of messages between two actors. The key characteristic of a discrete-event model is that each actor reacts to the input events in temporal order [50].

Lastly, in Figure 10, we also present the simulation results for the insulin pump model. The plot shows the historical consumption of insulin in the cartridge as consequence of the insulin doses administered (axis Y) over the simulation period (axis X).

#### 2.2.3. Stage 3—Validating the Device Model

The purpose of this stage is to evaluate the outputs (synthetic data) provided by the medical device model. This stage requires developers to instantiate the model via simulation tool providing all parameters for its execution. Thus, the inputs and outputs of the model are compared to check if the model behavior correctness and safety properties were achieved.

By analyzing the medical device models, we can observe that the sensor models usually have a simpler structure than the actuator models. This characteristic is related to the higher level for the safe operation of actuator devices such as insulin pump. The embedded software for sensor models are *discrete-event systems* and represent cyber world elements. They have a *local clock* that must be synchronized with the MCPS global clock and instantiated at its highest level of abstraction. Given this, it is possible to define the time interval in which the model collects continuous data from the patient.

To validate the insulin pump model, we simulate different input conditions to test all insulin administration strategies (e.g., *Basal* profiles, standard *Bolus* and corrective *Bolus*) and safety properties implemented in this model. An example of such safety properties is that the insulin pump must stop when the cartridge is completely empty or insufficient to administer the next scheduled insulin dose. Therefore, this stage requires one to define a clinical scenario for intended use of the insulin pump. Section 4.1 details the insulin pump model validation.

### 2.3. Model Library: Ready to be Reused

**Figure 11:**
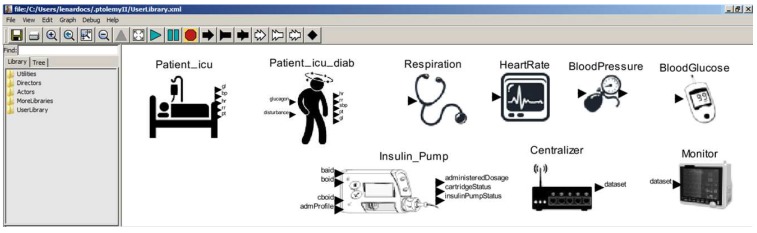
Model Library in Ptolemy II.

**Figure 12:**
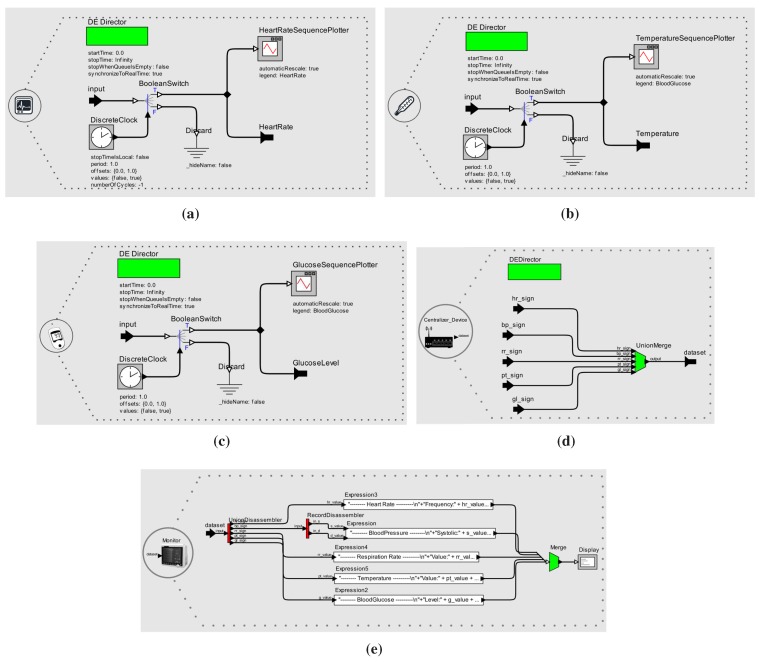
User Library. (**a**) Device Model for Cardiac Monitor; (**b**) Device Model for Thermometer; (**c**) Device Model for Glucometer; (**d**) Device Model for Data Centralizer; (**e**) Device Model for Bedside Monitor.

In practice, the model library becomes the user library in Ptolemy II, as illustrated in Figure 11 and Figure 12. Ptolemy II provides a *drag and drop* graphical interface to allow developers to easily reuse the models. The library has already two patient models (ICU and diabetic), five sensor models (cardiac and breathing monitors, blood pressure meter, thermometer and glucometer), one actuator (insulin pump) and two equipment models (bedside monitor and data centralizer).

Silva *et al.* [51] make available the XML file for all models, which can be opened by the Ptolemy II tool for building MCPS models using the guidelines described in Section 3.

## 3. Composition and Simulation: Early Validation of MCPS

**Figure 13:**
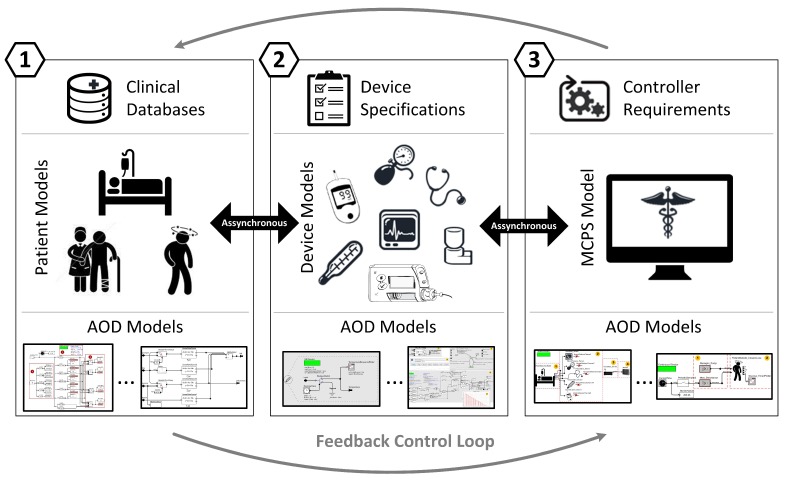
A Model-Based Architecture for MCPS.

In this section, we detail how to use the model library of patient and medical device formal models to build and simulate MCPS models according to the system requirements. MCPS models are responsible to control events and coordinate actions of medical devices and patients. An overview of the model-based architecture of an MCPS is illustrated in Figure 13. The architecture is composed of the following components: (i) Patient Models; (ii) Medical Device Models; and (iii) MCPS Model (controller). These models interact with each other to stimulate the reactions of other models. This interaction characterizes a *feedback control loop*. For instance, the medical sensor models monitor the patient model to capture the values of the vital signs periodically and to use them for decision making by the MCPS model. Afterwards, the MCPS model delegates the actions for the actuator models to execute changes in the patient model.

**Figure 14:**
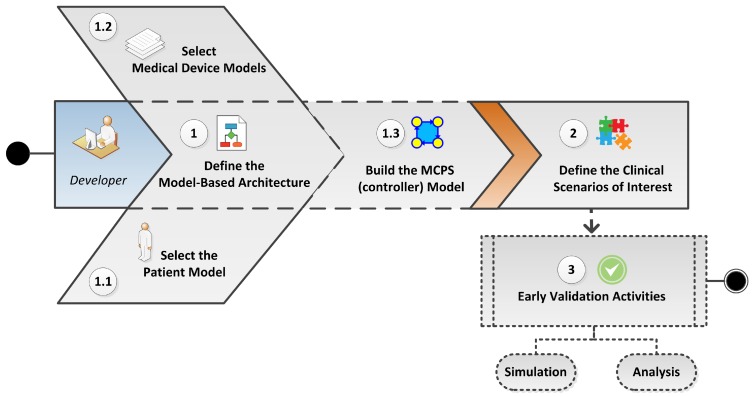
Model-Based Approach for Early Validation of MCPS.

As illustrated in Figure 14, our approach consists of: (1) designing the MCPS architecture, including selection of models for patients (1.1) and devices (1.2) and construction of the MCPS controller (1.3); (2) defining clinical scenarios of interest for simulation; and (3) performing model-based simulation and analysis. In the following subsections, we detail each phase of this process.

### 3.1. Designing the Model-Based Architecture

The architecture for the system being developed is a composition of patient and medical device models, integrated by the MCPS controller. The first step is to select the patient and device models from the library to be integrated to the MCPS controller. Both types of models are selected according to the system requirements. For instance, a developer that intends to build an MCPS for helping health experts to diagnose people with Asthma [52], a chronic lung disease that inflames and narrows the airways, needs to identify the primary symptoms of the disease, such as wheezing (a whistling sound when you breathe), chest tightness, shortness of breath, and coughing. The diagnosis of asthma is based on the patient medical and family histories, a physical exam, and test results. A test called spirometry is used to check how much air the patient can breathe in and out, and how fast she/he can blow air out. In this case, the patient model must incorporate the lung behavior, and the medical device model to be selected in the context of this system is the spirometer.

Other diseases will lead to other behaviors to be represented by the patient model, as well as to other medical device models for either the monitoring or actuation combined to the patient model. If there is no patient or device model available in the library for a given set of MCPS requirements, new models should be built and added to the library, according to the process described in Section 2.

After selecting the patient and device models, they must be integrated to compose the MCPS model. This process is detailed in the next subsections.

#### 3.1.1. Selecting Patient Models

The first stage is to select the patient model for the MCPS. The inherent complexity of a patient model described in Section 2 is hidden from the MCPS developer.

**Figure 15:**
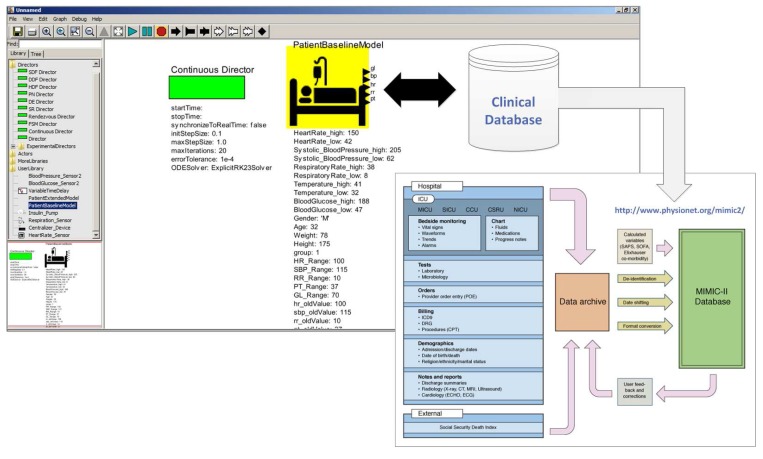
Selecting a Patient Model in the Ptolemy II.

As illustrated in Figure 15, the patient model is a reusable model in Ptolemy II user interface.

The selection of the patient model depends on the characteristics of the dataset required by the simulation scenarios to be evaluated by the developer. In this example, the patient model abstracts the behavior of patients in accordance with MIMIC II Clinical Database v2.6 [29]. This dataset contains clinical data from Intensive Care Unit (ICU) patients.

#### 3.1.2. Selecting Medical Device Models

**Figure 16:**
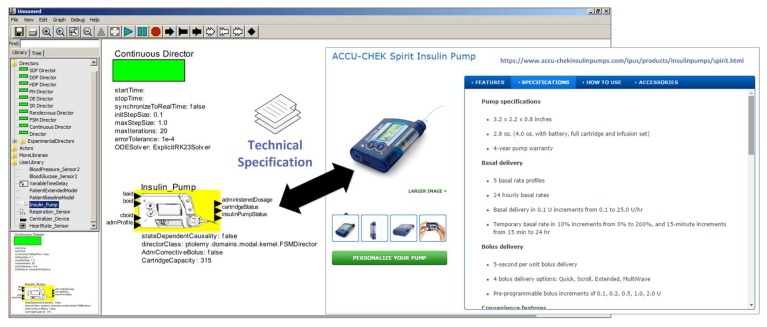
Selecting a Device Model in the Ptolemy II.

The selection of the device model depends on the technical requirements of the device demanded by the simulation scenarios to be evaluated by the developer. In the example illustrated in Figure 16, the model abstracts the behavior of an insulin pump.

#### 3.1.3. Composing the MCPS Model

The MCPS model controls events and coordinates actions of medical device and patient models to represent a real MCPS. After analyzing data from medical sensor models, an MCPS model delegates actions to the actuator models to administer drugs according to the patient health condition. The patient current state is given by the vital signs data generated by the patient model.

This idea of *feedback* in MCPS can be understood as the use of any available information about the system behavior to adjust the input to the system continuously [38]. This input is often viewed as a control signal that aims to achieve a desired behavior (*i.e.*, reference signal). Thus, the key property of feedback is to make corrections, especially when unexpected disturbances occur. A controlled system model that uses feedback definition allows the developer to implement two forms of control: (i) *Open-loop Control*, in which the input remains fixed regardless of the effect (good or bad) that it has on the observed output and (ii) *Closed-loop Control*, in which the input depends on the effect it causes on the output. This control logic depends on the features of each MCPS, and the developer is responsible to model the system control rules.

**Figure 17:**
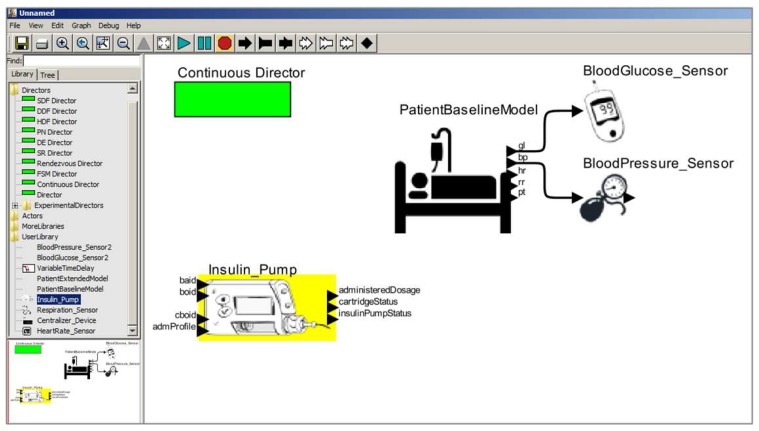
Defining an MCPS Model in the Ptolemy II.

The integration of patient and device models to the MCPS can also be done through the intuitive interface of Ptolemy II (Figure 17).

### 3.2. Defining the Clinical Scenarios of Interest

Developers must define clinical scenarios, which work as *simulation cases* used for analyzing system behaviors [49]. A *Clinical Scenario* can be used to simulate *Integrated Clinical Environments* (ICE), where the monitoring, diagnosis, and treatment of a patient are performed. ICE combine interoperable heterogeneous medical devices and other equipment to build a medical system to treat a high acuity patient [12]. Examples of ICE are operating rooms, intensive care units, field hospitals, ambulances, and other acute care environments.

The benefits of defining clinical scenarios independently from the medical devices include: (i) the ability to combine the data collected from different sources to yield new information; (ii) the clinical decision support; and (iii) the ability to implement distributed control of medical devices for safety interlocks and closed-loop control. We present three examples of clinical scenarios models in Section 4.1.

### 3.3. Running Early Validation Activities

Early validation activities consist in the definition of clinical scenarios (*i.e.*, use cases) that will serve to analyze the behavior of the MCPS model (Section 4.1), as well as of the mapping of their functional and safety requirements MCPS to a formal notation (Section 5). Such mapping aims to transform these requirements in formal properties that must be held for the different clinical scenarios. In addition, assumptions about the MCPS model can be made to simplify aspects of the model, and constraining input values for simulation purpose. With the simulation of different clinical scenarios it is possible to verify how the system reacts to several input conditions, and how much of the model’s structure is covered from these inputs. Thus, model coverage techniques can be used to identify unexecuted or unused simulation pathways in the model.

## 4. Validation of the Proposed Approach

In order to validate the proposed approach, we performed a two-step process. First, we applied it to three different clinical scenarios to evaluate its reusability potential for different contexts. Second, we performed an empirical evaluation with developers, applying the Goal-Question-Metric method, to evaluate early validation reusability and productivity.

### 4.1. Validating the Approach for Different Clinical Scenarios

We defined three proofs of concept to show the use of the models constructed by following the proposed approach. Each one represents a different clinical context, which could be used to support validation activities in an MCPS design. The MCPS models constructed include medical device models, other functional components, and patient models.

#### 4.1.1. Clinical Context I

In this scenario, we describe the situation in which patients are continuously monitored in an intensive care unit (ICU). During the stay of a patient in the ICU, their vital signs and physiological parameters are recorded. Such records are used to assess the patient health conditions over the treatment period.

**Figure 18:**
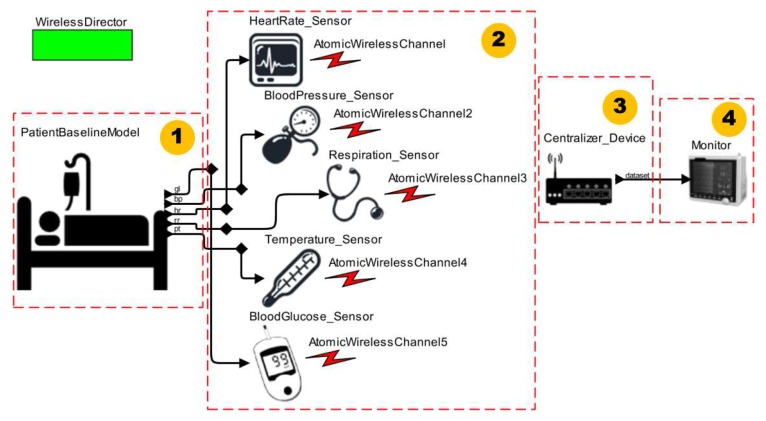
MCPS Model Using the ICU Patient Model.

The clinical scenario model is shown in Figure 18. We connect the medical sensor models **(2)** to the ICU Patient Model **(1)** via ports. This allows us to capture the data provided by the patient model. We construct a centralizer device model **(3)** to allow the communication between medical sensor models and a bedside monitor model **(4)**. Then, we define one communication channel in the centralizer model for each medical sensor model. The centralizer receives the medical sensor data through wireless interfaces, and makes them available to the bedside monitor model. The bedside monitor displays data for healthcare professionals and aids in decision-making.

**Figure 19:**
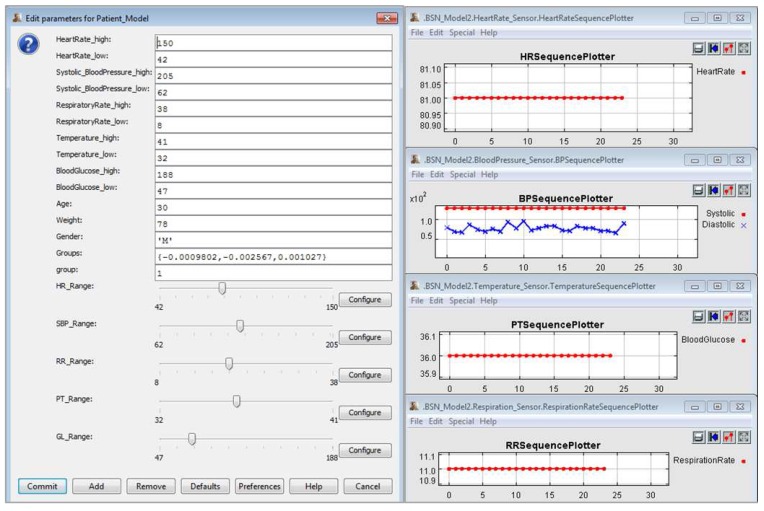
Simulation of an MCPS Model Using the ICU Patient Model.

In Figure 19, we show the screenshots with simulation data after execution of this MCPS model, and all configuration parameters for the ICU Patient Model and *Ptolemy II* simulator itself. This scenario allowed us to demonstrate that the developer has the flexibility to simulate various clinical conditions regarding the vital signs by using the ICU Patient Model. When this patient model is coupled to medical sensor models, we can monitor its data and use it to establish the treatment strategies for the patient. Thus, in this proof of concept we show how to apply the proposed approach in simpler clinical scenarios. In this case, the scenarios do not include actuator models.

#### 4.1.2. Clinical Context II

We defined this scenario to analyze the behavior of the insulin pump model when used by patients with diabetes mellitus. We chose this clinical scenario because it represents one of the main concerns of the World Health Organization (WHO), since diabetes is a chronic disease that affects about 347 million people of the world’s population [53]. It increases by 22% the risk of death from cardiovascular disease, and it is directly responsible for 3.5% of deaths in its group. Finally, diabetes can become the seventh leading cause of death in the world by 2030 [54].

In the context of this scenario, the patient will need to configure the insulin pump according to medical indications and his daily feeding regimen. Programming the insulin pump consists of selecting the *Basal* insulin administration profile and specifying the standard *Bolus* and corrective *Bolus* to be administered at specific times of the day. In the case of the corrective *Bolus*, patients activate a pump button to have a “shot” of insulin instantly injected into their body.

**Figure 20:**
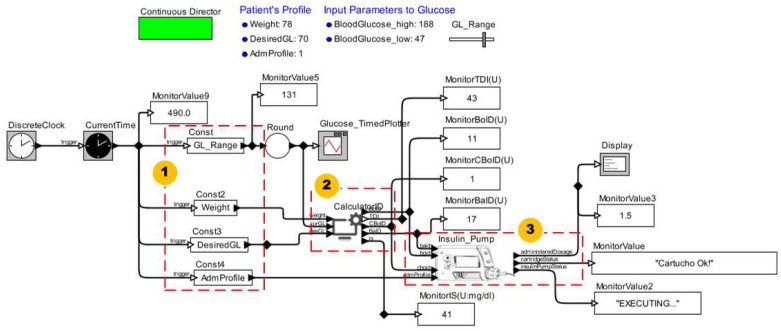
MCPS Model Using the Insulin Pump Model.

We present the model for this scenario in Figure 20. To execute the insulin pump model (3), it is necessary to input data such as configuration parameters (1). We obtain these parameters from the patient model. We model a functional component (2) that automatically calculates daily insulin doses according to the patient current health condition. The equations used to calculate such insulin doses are available in the clinical guidelines of the *Brazilian Society of Diabetes* [55].

To simulate the model of this clinical scenario, we specify the configuration parameters of the insulin pump model and patient profile. The *CalculatorID* component uses data of the patient profile in the equations. The outputs of the insulin pump model are the administered insulin rate and information about the operating states as well as cartridge level. The results of these simulations are similar to the chart presented in Figure 8.

This proof of concept was relevant to verify safety properties implemented in the insulin pump model and ensure compliance with the specifications. We executed this model twice. In the first simulation we set the *Basal* administration profile for the standard mode (*i.e.*, fixed doses), and in the second in customized mode (*i.e.*, flexible doses). For both simulations, we have not verified any violations to the insulin pump’s safety properties or even failures during its operation.

#### 4.1.3. Clinical Context III

In this scenario, we use insulin pharmacokinetic to demonstrate how to use the Diabetic Patient Model. There are two distinct strategies to use the Diabetic Patient Model in this clinical context. First, to control the glucose level as a closed-loop system, in which a proportional-integral-derivative (PID) controller is designed for this purpose and embedded in the patient model (intrusive). We presented this process in Section 2.1. The alternative way is to control the glucose level by means of the insulin pump model coupled to the patient model (non-intrusive), as an open-loop system.

**Figure 21:**
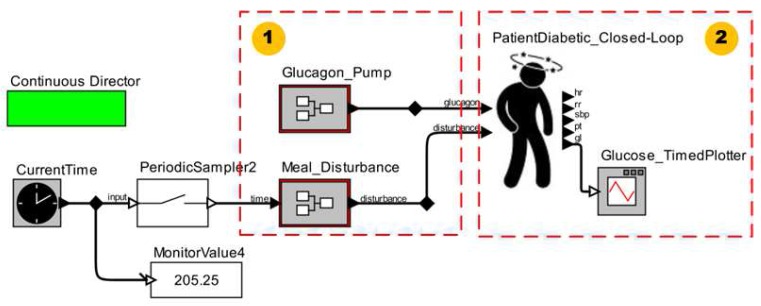
MCPS Model Using a Diabetic Patient Model.

In Figure 21, we show the MCPS model for the first strategy (*i.e.*, closed-loop system). We observe in this scenario model the presence of only functional component models (1) to generate the disturbances for the patient model (2).

**Figure 22:**
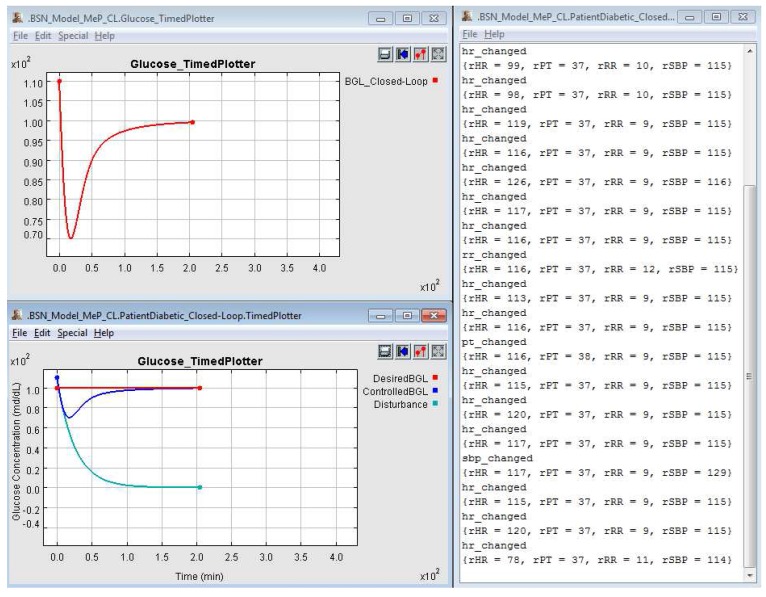
Simulation of an MCPS Model Using the Diabetic Patient Model.

The simulation results of this clinical scenario model is shown in Figure 22. The patient model behavior represents the same response regarding the glucose if the patient model receives disturbances. Therefore, this patient model reacts as shown in the end of the ICU Patient Model. Thus, during the simulation of this scenario we noticed that the glucose control method induces the regression models to adjust the respective values for each vital sign. This occurs because such regression models use the glucose (physiological parameter) as one predictor variable.

In this scenario, the values for the vital signs can be changed during simulation, not only by the developers who want to simulate abnormal situations regarding the vital signs, but also by the PID controller, which acts to control the glucose level in response to disturbances.

### 4.2. Empirical Evaluation

To evaluate if our approach promotes reusability and productivity, we performed an empirical study. The study was performed considering developers’ viewpoint. For this purpose, we evaluated the library of patient and medical device models with fourteen software developers. The evaluation is divided into two phases: training and evaluation. During the training phase, a trainer prepared the developers to build MCPS using Ptolemy II. During the evaluation phase, the developers were divided into two groups: control and treatment. The control group developed two MCPS models with only a small subset of the library of patient and medical device models available. The treatment group developed the same MCPS models with the entire library available. Productivity was assessed by, for each MCPS, applying the *t*-test with the mean time that each group took to finish implementing it. Afterwards, the developers responded a survey regarding model reusability indicators: understandability, adaptability and portability.

#### 4.2.1. Scoping

The goals of the empirical evaluation, using the Goal-Question-Metric (GQM) goal template presented by Basili and Rombach [24], are: (i) analyze *the usage of the library of patient and medical device models* for the purpose of *evaluation* with respect to *productivity* from the viewpoint of the *developer* in the context of *developers building MCPS models*; and (ii) analyze *the usage of the library of patient and medical device models* for the purpose of *evaluation* with respect to *reusability* from the viewpoint of *the developer* in the context of *developers building MCPS models*. We addressed the following research questions:

***RQ1*** Does using the library of patient and medical device models increase developers’ productivity?
***RQ2*** Are the library’s models reusable?

Given the research questions, we defined the following informal hypotheses:

***H0-1*** Productivity is not increased.
***HA-1*** Productivity is increased.
***H0-2*** The models are not reusable.
***HA-2*** The models are reusable.

In which, ***H0-1*** and ***HA-1*** are related to ***RQ1*** and ***H0-2*** and ***HA-2*** to ***RQ2***.

#### 4.2.2. Objects of study

According to Kitchenham, Pickard and Pfleeger [56], objects of study may be the programs, algorithms, or problems to which the methods or tools are applied in a given experiment. In this research, we defined two problems to analyze the usage of the library of patient and medical device models: (i) build the MCPS model for the clinical scenario presented in Section 4.1.1, in which patients are continuously monitored in an intensive care unit (ICU); and (ii) validate the behavior of the insulin pump model presented in Section 4.1.2.

#### 4.2.3. Subjects

We used the convenience sampling technique. The empirical evaluation was executed in a Brazilian company that was chosen given existing academia-industry relations. As subjects, the company provided a group of fourteen developers. The developers’ profile is shown in Table 4.

**Table 4:** Questionnaire to Identify the Developer’s Profile.

Questions	Developers													
	1	2	3	4	5	6	7	8	9	10	11	12	13	14
*Years Old*	21	25	20	21	30	21	24	27	21	21	20	25	22	23
*Knowledge on Formal Methods?* ${}^{b}$	Y	Y	N	N	Y	Y	N	Y	N	Y	Y	Y	Y	Y
*Opinion About the Training Phase?* ${}^{a}$	GREAT	NORMAL	GOOD	GREAT	GOOD	GREAT	GREAT	GREAT	GREAT	GREAT	GOOD	GOOD	GOOD	GREAT
*Resolved the list of exercises?* ${}^{a}$	Y	Y	Y	Y	N	Y	Y	Y	Y	Y	Y	N	Y	N
*Knowledge of Ptolemy II?* ${}^{a}$	GOOD	GOOD	GOOD	NORMAL	LITTLE	GOOD	NORMAL	NORMAL	NORMAL	GOOD	NORMAL	NORMAL	GOOD	GOOD
*Knowledge About Our Work?* ${}^{a}$	GOOD	NORMAL	GOOD	NORMAL	LITTLE	GOOD	NORMAL	NORMAL	GOOD	GOOD	NORMAL	GOOD	GOOD	GOOD

^*a*^ After the Training Phase; ^*b*^ Before the Training Phase; Y: Yes; N: No.

#### 4.2.4. Variables and Treatment

Given the research goals, we have two dependent (*i.e.*, response) variables: developers’ productivity and models’ reusability. Furthermore, we have four independent variables (*i.e.*, factors): the library of patient and medical device models, the experience of the personnel (*i.e.*, subjects), tool support and the environment. The last three factors are controlled at a fixed level. All subjects have the same experience, tool support and environment. Therefore, the treatment factor is *the library of patient and medical device models*.

#### 4.2.5. Procedure

After Ptolemy II has been installed and a working environment for each developer has been setup, the study was executed in two phases: training and evaluation. These phases are described in what follows.

**Training phase**

During this phase, we prepared the subjects to develop MCPS models in Ptolemy II using the library of patient and medical device models. The following activities were planned:

- **Personal questionnaire**—developers answer a questionnaire regarding personal information, experience with formal methods and experience with components reusability.
- **Presentational learning**—one of the researchers (*i.e.*, trainer) gives an introductory course in which concepts regarding MCPS, Ptolemy II and the proposed method are presented. Furthermore, working examples of patient and device models in Ptolemy II are shown.
- **Autonomous hands-on learning**—(*i.e.*, learning by doing) with online help from the trainer. The subjects applied the learned techniques to build simple elements in Ptolemy II such as an incremental counter and a sinusoidal signal sensor.

The training material (e.g., slides, Ptolemy II example files) were prepared by the trainer. During the introductory course, audio-visual presentations were used. For supporting the hands-on learning activities, we used the individual problem-solving method.

**Evaluation phase**

During this phase, the subjects used the library of patient and medical device models to build MCPS models in Ptolemy II and responded a questionnaire regarding the models’ reusability attributes. The following activities were planned:

- **MCPS modeling**—the developers were divided into two balanced (*i.e.*, same size) groups: control and treatment. Each group was composed of seven subjects selected randomly. The control group solved the two problems presented in Section 4.2.2 using only a small subset of the library of patient and medical device models. The treatment group solved the same problems using the entire library, except the centralizer device. Therefore, we used an experiment design of type “blocked subject-object study”. For the control group, we provided the heart and respiratory rate monitors, and glucometer completely ready for reuse, as well as the ICU patient and Insulin Pump models partially ready for reuse. Furthermore, for both groups, a guideline was provided to assist on the MCPS modeling.
- **Reusability questionnaire reply**—the developers responded to a questionnaire regarding the models’ reusability attributes: understandability, adaptability and portability.

The evaluation material (e.g., Ptolemy II files, questionnaire) were prepared by the researchers. During the MCPS modeling, the trainer was available to help with any misunderstanding on the clinical scenarios definitions and questionnaire wording.

#### 4.2.6. Measures

To evaluate the hypotheses, assess the research questions and research goals, we collected metrics. Furthermore, by defining the metrics, we formalized the hypotheses presented in Section 4.2.1 to statistically test them. As stated in Section 4.2.4, we have two dependent variables: developers’ productivity and models’ reusability.

**Measuring productivity**—according to Kitchenham, Pickard and Pfleeger [56], to measure productivity, it is necessary to address effort and time. In the *MCPS development* activity, the control and treatment groups developed the MCPS models for the same clinical scenarios. The difference was that the treatment group had more models from the library available for reuse. Therefore, we only measured the time required by each group to finish the problems. Whenever each subject finished an MCPS model, the time required to finish it was collected. Therefore, for each problem, we formally defined a null and an alternative hypothesis:

***H0-1-1***  $\mu_{C_{1}}\leq \mu_{T_{1}}$, in which $\mu_{C_{1}}$ is, for the first problem, the mean required time (in minutes) by the control group to finish it, and $\mu_{T_{1}}$, by the treatment group.

***HA-1-1***  $\mu_{C_{1}}>\mu_{T_{2}}$.

***H0-1-2***  $\mu_{C_{2}}\leq \mu_{T_{2}}$, in which $\mu_{C_{2}}$ is, for the second problem, the mean required time (in minutes) by the control group to finish it, and $\mu_{T_{2}}$, by the treatment group.

***HA-1-2***  $\mu_{C_{2}}>\mu_{T_{2}}$.

These hypotheses were evaluated using the t-test to assess ***RQ1***.

**Measuring reusability**—according to Washizaki, Yamamoto and Fukazawa [57], component reusability can be decomposed into three factors: understandability, adaptability and portability. Understandability is the effort needed by a user to recognize the concept behind a component and its applicability. Adaptability is the ease with which a component can be adapted to fulfill a requirement that differs from that for which it was originally developed. Portability is the ease with which software can be transferred from one environment to another. As Washizaki, Yamamoto and Fukazawa, we want a black-box evaluation of the component from the viewpoint of the users. Therefore, we discard well-known reusability criterion such as cohesion and coupling and focus on the three factors previously described.

To collect metrics regarding these factors, we created a questionnaire and, for each question, we provided a 5-point Likert scale from worst (1) to best (5). For instance, for understandability, the questionnaire has the question “How do you rate the effort needed to recognize the concept behind a component and its applicability?” and the answer 5 represents “Very little effort”. At the end of the *MCPS models development* activity, the subjects responded the questionnaire. Therefore, for each factor, we defined a null and an alternative hypothesis:

***H0-2-1***  $\upsilon_{U}\leq 3$, in which $\upsilon_{U}$ represents the mean rank for the answers for the question regarding understandability.

***HA-2-1***  $\upsilon_{U}>3$.

***H0-2-2***  $\upsilon_{A}\leq 3$, in which $\upsilon_{A}$ represents the mean rank for the answers for the question regarding adaptability.

***HA-2-2***  $\upsilon_{A}>3$.

***H0-2-3***  $\upsilon_{P}\leq 3$, in which $\upsilon_{P}$ represents the mean rank for the answers for the question regarding portability.

***HA-2-3***  $\upsilon_{P}>3$.

These hypotheses were evaluated using the Wilcoxon test to assess ***RQ2***.

#### 4.2.7. Analysis

***RQ1*** Does using the library of patient and medical device models increase developers’ productivity?

**Figure 23:**
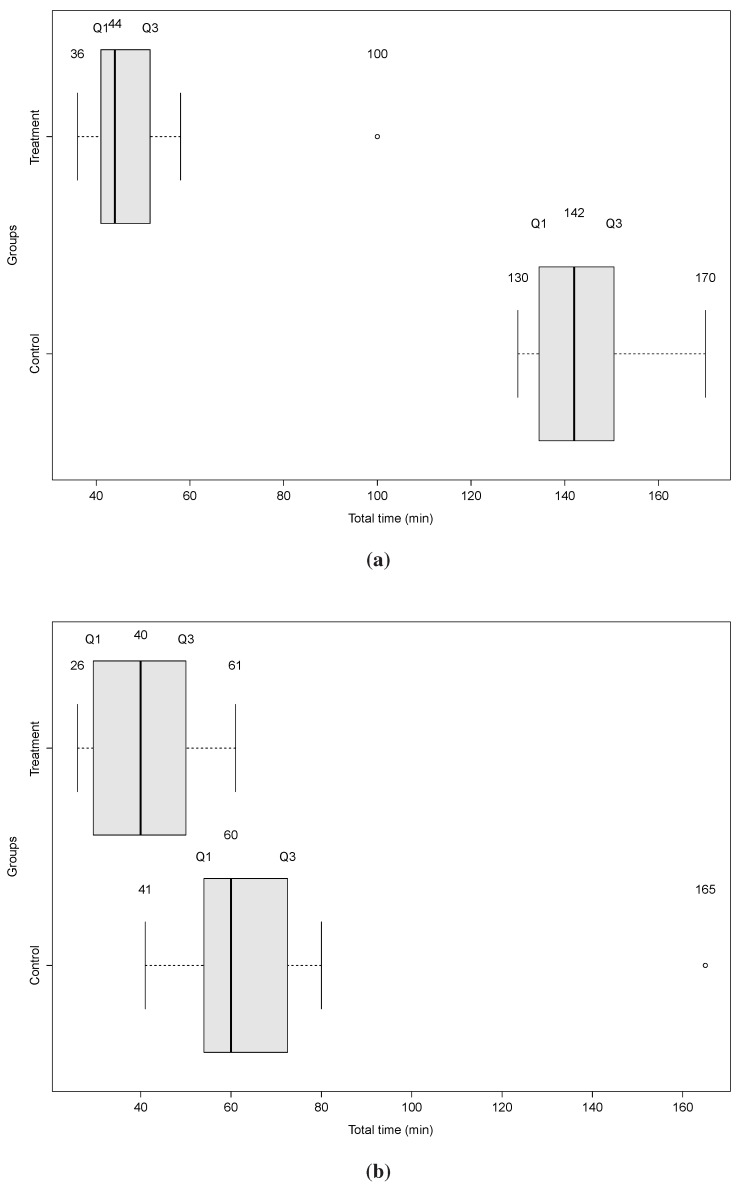
Boxplots of the Time Collected for the Control and Treatment Group. (**a**) First Problem—Clinical Context I.; (**b**) Second Problem—Clinical Context II.

To assess this research question, we evaluated ***H0-1-1*** and ***H0-1-2*** using the t-test with 95% confidence interval for the difference between the two group means. In Figure 23, we show, for each problem, the time collected for the control and treatment group.

For ***H0-1-1***, we had a *p-value* of 1.41e−06 and refuted the null hypothesis. This means that the control group has a mean statistically greater than the treatment group (at the 0.05 significance level). In practice, the treatment group saved much time (*i.e.*, approximately three times less) to model the Clinical Scenario I than the control group.

For ***H0-1-2***, we had a *p-value* of 0.04181 and refuted the null hypothesis. In this case, the treatment group saved half of the time required to model the Clinical Scenario II compared to the control group. This reduction in the difference between the two group means occurred due to the reduced number of steps necessary to model this second scenario.

Therefore, we conclude that the reuse of the patient and device models available in our model library increases the productivity of the developers during the MCPS early validation.

***RQ2*** Are the models built with the proposed approach reusable?

**Figure 24:**
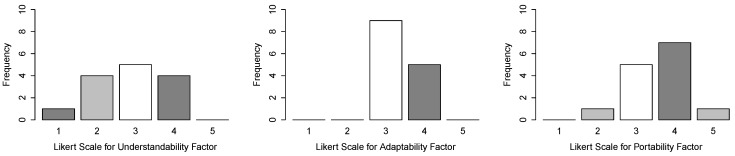
Answers Distribution in Accordance with the Reusability Factors.

To assess this research question, we evaluated ***H0-2-1***, ***H0-2-2*** and ***H0-2-3*** using the Wilcoxon test with 95% confidence interval for the difference between the response of all developers and the parameter specifying the value used to form the null hypothesis, where this parameter is equal to three. Thus, such parameter defines the threshold to say if the model is reusable or not. In Figure 24, we depict the answers for each reusability factor.

For ***H0-2-1***, we had a *p-value* of 0.7394 and accept the null hypothesis. Since most of the developers have opined that the training phase was great (*i.e.*, about 53% the developers) with a good knowledge acquired about our work (*i.e.*, about 60% the developers), the reason for the test result of this hypothesis may be the little experience of the developers with Ptolemy II framework. This means that the exercise list practiced by the developers (*i.e.*, about 80%) during the training phase was insufficient for them to model the clinical scenarios requested in the evaluation stage.

For ***H0-2-2***, we had a *p-value* of 0.01844 and refuted the null hypothesis. In practice, we can say that the patient and device models available in our model library are easily adaptable to fulfill a requirement of other clinical contexts.

For ***H0-2-3***, we had a *p-value* of 0.01238 and refuted the null hypothesis. Thus, we can say that the patient and device models available in our model library are portable from one specific clinical scenario to another.

Despite the reported problem in the hypothesis testing for ***H0-2-1***, we conclude, at 0.05 significance level, that the patient and device models available in our model library are reusable to support the developers in the MCPS validation.

#### 4.2.8. Threats to Validity

Conclusion validity is concerned with the relationship between the treatment and the outcome. We mitigated most of the conclusion validity such as heterogeneity of subjects, reliability of treatment implementation and reliability of measures. On the other hand, our sample size (fourteen subjects divided into two groups of seven) was small. Therefore, we used a t-test with 0.05 significance.

Internal validity is of concern when causal relationships are examined. In our case, the issues are related to Ptolemy II. The tool has some defects related to saving the models and executing simulations that might have influenced the time required by the subjects to implement the clinical scenarios. For instance, during the drag and drop of a model from user library to the graph editor, its internal structure was shuffled, making impossible to understand the model. In this case, the developer had to keep trying to copy it until this behavior does not happen.

External validity is concerned with to what extent it is possible to generalize the findings, and to what extent the findings are of interest to other people outside of the investigated context. The empirical study was executed with subjects from one company and with little or no experience building MCPS. Given that all the subjects were from one company, we cannot generalize our findings. Furthermore, on one hand, having the subjects from the treatment group (almost completely reusing the library) successfully implementing the scenarios is a good indicator of the library’s reusability potential. On the other hand, one could argue that the productivity gain would not be significant if experienced MCPS and Ptolemy II developers participated as subjects.

Construct validity is concerned with the relation between theory and observation. For objects of study, we used two clinical scenarios. This might not be enough to represent the theory.

## 5. Model Analysis

To analyze the models that define the clinical scenarios presented in Section 4.1, we used *Simulink Design Verifier* (SLDV) [25]. SLDV is an optional component of Matlab^®^, which enables model analysis within the Simulink environment. It uses formal methods to identify hard-to-find design errors in models, including dead logic, integer overflow, division by zero, and violations of design properties and assertions. As a result, we can perform verification and validation throughout the design process. Thus, model analysis with SLDV complements simulation by allowing simulation results as inputs to analysis with formal methods.

**Figure 25:**
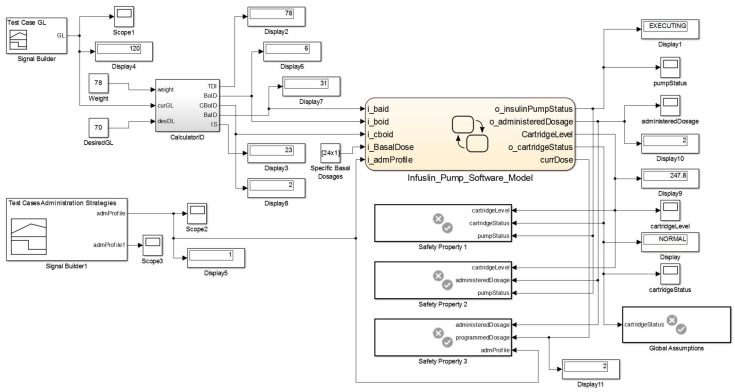
MCPS Model Using the Insulin Pump Model in Simulink.

In this article, we demonstrate the process that we applied to verify the insulin pump software model shown in Section 2.2 and used in the scenario of the second clinical context, as described in Section 4.1.2. Since *Ptolemy II* framework does not support formal verification, to perform safety analysis on the insulin pump software model we constructed the MCPS model in Simulink (see Figure 25).

**Figure 26:**
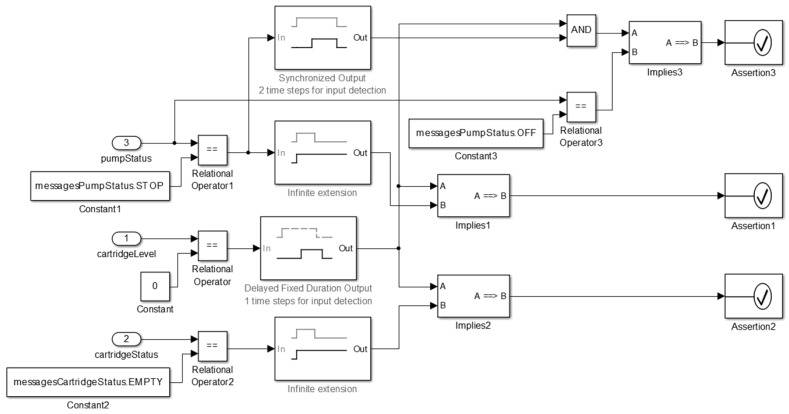
Safety Property 1.

**Figure 27:**
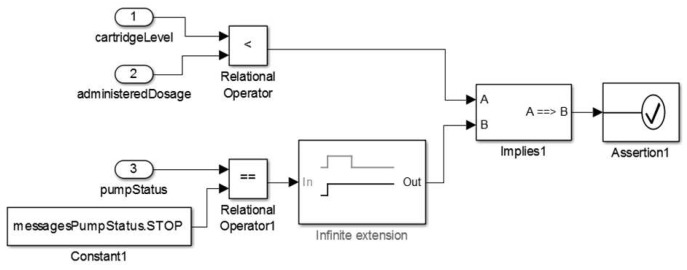
Safety Property 2.

**Figure 28:**
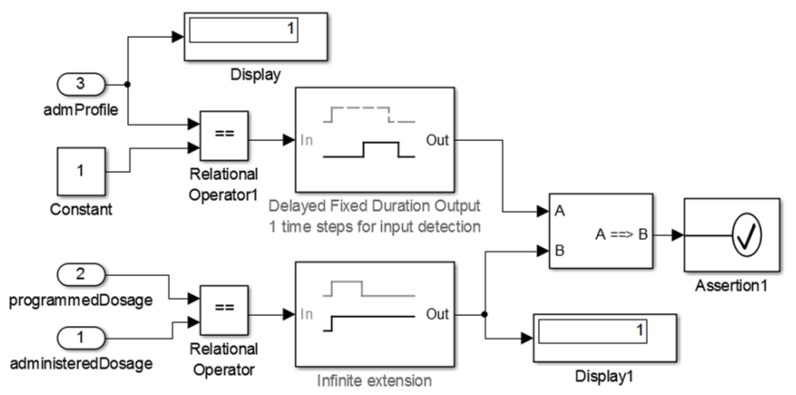
Safety Property 3.

Afterwards, we verified basic safety properties. With this purpose, we formalized safety requirements as safety properties. Safety properties are unwanted behaviors that should never occur or functional requirements that must be satisfied. For example, for insulin pump model, we defined the following three safety requirements:

*Safety Requirement 1 (SR1):*  if cartridge’s level is equal to 0, then the cartridge’s status shall become EMPTY and after a delay the pump’s status shall be STOP. The formalization of this property is shown in Figure 26.
*Safety Requirement 2 (SR2):*  if cartridge’s level is lower than the administered insulin dosage then the pump’s status shall be STOP. The formalization of this property is shown in Figure 27.
*Safety Requirement 3 (SR3):*  whenever the administration profile becomes 1 (SPEC_BASAL) the administered dosage shall equal the programmed dosage. The formalization of this property is shown in Figure 28.

**Figure 29:**
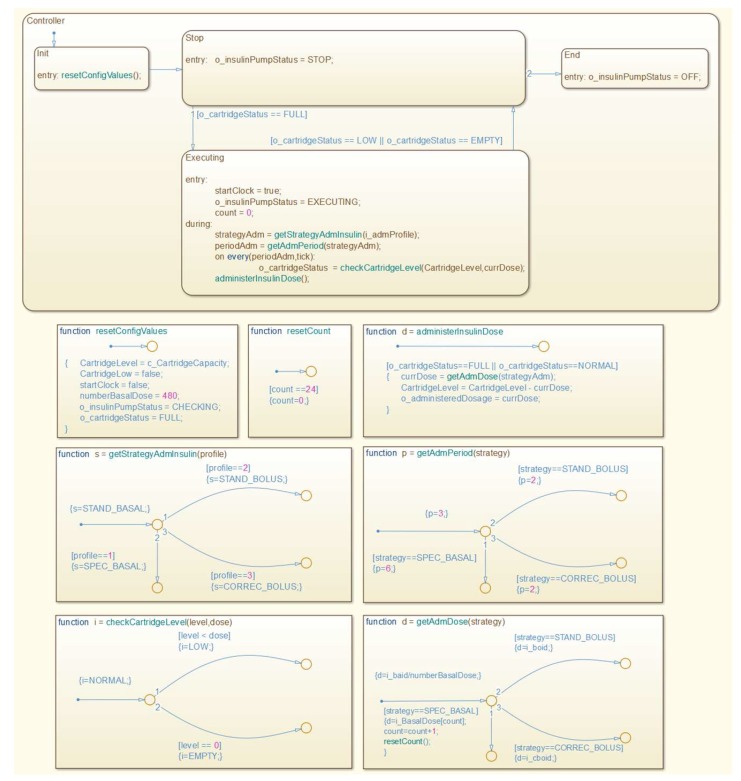
Insulin Pump Software Model in Simulink.

We represented the behavior of the insulin pump software with a Stateflow [58] diagram used in conjunction with Simulink, as shown in Figure 29. The Stateflow is an environment for modeling and simulating combinatorial and sequential decision logic based on state machines and flow charts.

**Figure 30:**
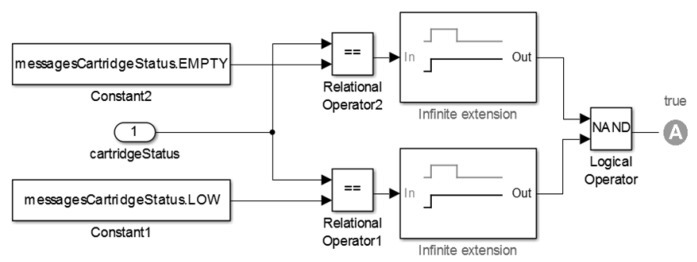
Assumption About the Cartridge’s Status.

We defined an insulin pump software constraint with the following assumption (A1): the cartridge’s status shall never be EMPTY and LOW simultaneously. We formally modeled this assumption as shown in Figure 30.

To prove SP1, it is necessary to satisfy three proof objectives that are specified as assertions. To prove SP2, SP3 and A1, it is necessary to satisfy one proof objective. We used SLDV to validate all the requirements and constraints. The mean elapsed time for the analysis in MATLAB *R2013a* was 160 s; we used a PC Intel(R) Core(TM) i5-3210M CPU @ 2.50GHz with 6GB RAM and Windows 7 operating system.

We analyzed the insulin pump software model coverage through SLDV, which accumulates information about activation of verification objectives during simulation and provides coverage results [25]. From the Simulink and Stateflow models, the SLDV generates test cases and parameters required by industry standards for developing high-integrity systems. The structural coverage criteria used in this analysis were decision (DC), condition (CC), and modified condition/decision coverage (MCDC) [59]. For DC, the test suite must have tests that verifies each reachable decision as true and false. Thus, the DC analyzes elements that represent decision points in a model, such as a Simulink switch block or Stateflow states. For CC, the test suit must have tests that verify each condition in the program as true and false. Therefore, the CC analyzes blocks that output the logical combination of their inputs (e.g., the logical operator block) and Stateflow transitions. Lastly, the MCDC criteria requires that the condition *c* independently affects the outcome of the decision *d*. It combines the decision and condition capabilities to determine the extent to which the test case verify the independence of logical block inputs and transition conditions.

We present the summary of the model coverage results for the Insulin Pump Software Model subsystem in Table 5.

We achieved 100% DC for the objects *Controller*, *administerInsulinDose*, *getAdmPeriod*, *getStrategyAdmInsulin*, and *resetCount* object. We achieved less than 100% coverage of the decisions analyzed for *checkCartridgeLevel* and *getAdmDose*. The *checkCartridgeLevel* achieves 50% decision coverage because the transition “[level == 0]{i=EMPTY;}” never evaluates to true during simulation with the test case provided as input for the insulin pump software model. The same occurred with the transitions “[strategy==STAND_BOLUS]{d=i_boid;}” and “[strategy==CORREC_BOLUS]{d=i_cboid;}” of *getAdmDose*.

**Table 5:** Summary of the Coverage Report for Insulin Pump Software Model.

Model Hierarchy	Cyclomatic Complexity ${}^{\left(1\right)}$	DC	CC	MDCC
Subsystem:Insulin Pump Software Model	22	92%	75%	50%
Chart:Insulin Pump Software Model	21	92%	75%	50%
State:Controller	7	100%	75%	50%
Function:administerInsulinDose	2	100%	75%	50%
Function:checkCartridgeLevel	2	75%	NA ${}^{\left(2\right)}$	NA
Function:getAdmDose	3	67%	NA	NA
Function:getAdmPeriod	3	100%	NA	NA
Function:getStrategyAdmInsulin	3	100%	NA	NA
Function:resetCount	1	100%	NA	NA

${}^{\left(1\right)}$ Measure of the structural complexity for each object in the model hierarchy; ${}^{\left(2\right)}$ NA: not applicable.

We achieved 75% of CC because the following conditions never evaluate to true: (1) “o_cartridgeStatus == EMPTY” of the transition “[o_cartridgeStatus == LOW || o_cartridgeStatus == EMPTY]” from substate *“Executing”* to *“Stop”*; (2) “o_cartridgeStatus == FULL” of the transition “o_cartridgeStatus == FULL || o_cartridgeStatus == NORMAL]”. Both transitions also affect the MCDC analysis causing the model to achieve only 50% coverage. In this case, the reason is that the specified combination of inputs represented in such transitions did not occur during the test case included in the report. Given this, the Insulin Pump Software Model achieved 92% coverage.

## 6. Conclusions

In this article, we presented an approach for model-based early validation of Medical Cyber-Physical Systems (MCPS). We introduced a reusable model library composed of two types of models: patients and devices. Patient models are based on medical guidelines and clinical databases. Device models are built from the technical specification of sensor devices and actuators. The models are built as reusable components, being customizable enough to be reused in different MCPS.

We demonstrated the practical viability of the solution by creating models for three different clinical contexts: (1) an intensive clinical unit environment; (2) usage situations of an insulin infusion pump; and (3) a diabetic patient context that needs to control the blood glucose level. We also successfully validated our approach through an empirical evaluation with developers. Finally, we performed a formal verification of the models, checking functional and safety requirements as well as model coverage of one of the three clinical scenarios presented.

As future work, we plan to extend our approach to late validation of MCPS. In other words, we intend to integrate our composition and simulation guidelines with approaches for simulation and testing of device hardware and software. More specifically, it is possible to apply Hardware In the Loop (HIL) [60] mechanisms to allow integrating real medical device input-output to the AOD model, at runtime. In addition, we can generate test cases for certifying software systems and applications based on the simulation cases described in this article.

## References

[1] Arney D., Plourde J., Schrenker R., Mattegunta P., Whitehead S.F., Goldman J.M. (2014). Design Pillars for Medical Cyber-Physical System Middleware. OpenAccess Ser. Inform..

[2] Haque S.A., Aziz S.M., Rahman M. (2014). Review of Cyber-Physical System in Healthcare. Int. J. Distrib. Sens. Netw..

[3] Lee I., Sokolsky O., Chen S., Hatcliff J., Jee E., Kim B., King A., Mullen-Fortino M., Park S., Roederer A. (2012). Challenges and research directions in medical cyber-physical systems. IEEE Proc..

[4] Lee E. (2015). The Past, Present and Future of Cyber-Physical Systems: A Focus on Models. Sensors.

[5] Delaune S.C., Ladner P.K. (2011). Fundamentals of Nursing: Standards & Practice.

[6] Rosdahl C.B., Kowalski M.T. (2012). Textbook of Basic Nursing.

[7] González J.V., Arenas O.A.V., González V.V. (2012). Semiología de los Signos Vitales: Una Mirada Novedosa a un Problema Vigente. Arch. Med..

[8] FDA CFR—Code of Federal Regulations Title 21—Part 820. http://www.accessdata.fda.gov/scripts/cdrh/cfdocs/cfcfr/CFRSearch.cfm?CFRPart=820.

[9] Li T., Tan F., Wang Q., Bu L., Cao J.-N., Liu X. (2014). From Offline toward Real Time: A Hybrid Systems Model Checking and CPS Codesign Approach for Medical Device Plug-and-Play Collaborations. IEEE Trans. Parallel Distrib. Syst..

[10] Hassine J. (2015). Early modeling and validation of timed system requirements using Timed Use Case Maps. Requir. Eng..

[11] FDA Medical Device Recall Report—FY2003 to FY2012.

[12] ASTM International ASTM F2761-09(2013). http://www.webcitation.org/6ZdV3vBC1.

[13] Pajic M., Mangharam R., Sokolsky O., Arney D., Goldman J., Lee I. (2012). Model-Driven Safety Analysis of Closed-Loop Medical Systems. IEEE Trans. Ind. Inform..

[14] Jiang Z., Pajic M., Mangharam R. (2012). Cyber-Physical Modeling of Implantable Cardiac Medical Devices. IEEE Proc..

[15] Jiang Z., Pajic M., Alur R., Mangharam R. (2014). Closed-loop verification of medical devices with model abstraction and refinement. Int. J. Softw. Tools Technol. Transf..

[16] Miller B., Vahid F., Givargis T. Digital Mockups for the Testing of a Medical Ventilator. Proceedings of the 2nd ACM SIGHIT International Health Informatics Symposium (IHI).

[17] Van Heusden K., Dassau E., Zisser H.C., Seborg D.E., Doyle F.J. (2012). Control-Relevant Models for Glucose Control Using A Priori Patient Characteristics. IEEE Trans. Biomed. Eng..

[18] Kang W., Wu P., Rahmaniheris M., Sha L., Berlin R.B., Goldman J.M. Towards organ-centric compositional development of safe networked supervisory medical systems. Proceedings of the IEEE 26th International Symposium on Computer-Based Medical Systems (CBMS).

[19] King A.L., Feng L., Sokolsky O., Lee I. Assuring the safety of on-demand medical cyber-physical systems. Proceedings of the IEEE 1st International Conference on Cyber-Physical Systems, Networks, and Applications (CPSNA).

[20] Simalatsar A., de Micheli G. Medical guidelines reconciling medical software and electronic devices: Imatinib case-study. Proceedings of the IEEE 12th International Conference on Bioinformatics Bioengineering (BIBE).

[21] Li C., Raghunathan A., Jha N.K. (2013). Improving the Trustworthiness of Medical Device Software with Formal Verification Methods. IEEE Embed. Syst. Lett..

[22] Murugesan A., Sokolsky O., Rayadurgam S., Whalen M., Heimdahl M., Lee I. Linking abstract analysis to concrete design: A hierarchical approach to verify medical CPS safety. Proceedings of the ACM/IEEE 5th International Conference on Cyber-Physical Systems (ICCPS).

[23] Agha G. (1986). Actors: A Model of Concurrent Computation in Distributed Systems.

[24] Basili V.R., Rombach H.D. (1988). The TAME project: Towards improvement-oriented software environments. IEEE Trans. Softw. Eng..

[25] Mathworks (2012). Simulink Design Verifier.

[26] Berkeley U.C. The Ptolemy Project: Heterogeneous, Modeling and Design.

[27] Katzung B.G., Katzung B.G., Masters S.B., Trevor A.J. (2012). Introduction. Basic & Clinical Pharmacology.

[28] Hair J.F., Black W.C., Babin B.J., Anderson R.E., Tatham R.L. (2009). Análise Multivariada de Dados.

[29] PHYSIONET MIMIC II Databases, National Institutes of Health (NIH), 2009. http://physionet.org/mimic2.

[30] ADA (2004). Diagnosis and Classification of Diabetes Mellitus. Diabetes Care.

[31] Buxton A.E., Calkins H., Callans D.J., DiMarco J.P., Fisher J.D., Greene H.L., Haines D.E., Hayes D.L., Heidenreich P.A., Miller J.M. (2006). ACC/AHA/HRS 2006 Key Data Elements and Definitions for Electrophysiological Studies and Procedures: A Report of the American College of Cardiology/American Heart Association Task Force on Clinical Data Standards (ACC/AHA/HRS Writing Committee to Develop Data Standards on Electrophysiology). J. Am. Coll. Cardiol..

[32] Chobanian A.V., Bakris G.L., Black H.R., Cushman W.C., Green L.A., Izzo J.L., Jones D.W., Materson B.J., Oparil S., Wright J.T. (2003). Seventh Report of the Joint National Committee on Prevention, Detection, Evaluation, and Treatment of High Blood Pressure. Hypertension.

[33] Handelsman Y., Mechanick J., Blonde L., Grunberger G., Bloomgarden Z.T., Bray G.A., Dagogo-Jack S., Davidson J.A., Einhorn D., Ganda O. (2011). American Association of Clinical Endocrinologists Medical Guidelines for Clinical Practice for Developing a Diabetes Mellitus Comprehensive Care Plan: Executive Summary. Endocr. Pract..

[34] McGee S.R. (2007). Evidence-Based Physical Diagnosis.

[35] NHBPEP (2004). The Fourth Report on the Diagnosis, Evaluation, and Treatment of High Blood Pressure in Children and Adolescents. Pediatrics.

[36] Jain R. (1991). The Art of Computer Systems Performance Analysis: Techniques for Experimental Design, Measurement, Simulation, and Modeling.

[37] McCullagh P., Nelder J.A. (1989). Generalized Linear Models.

[38] Cassandras C.G., Lafortune S. (2008). Introduction to Discrete Event Systems.

[39] Mathworks PID Controller, Discrete PID Controller. http://www.mathworks.com/help/simulink/slref/pidcontroller.html.

[40] Mathworks MATLAB. http://www.mathworks.com/products/matlab/.

[41] Bergman R.N., Phillips L.S., Cobelli C. (1981). Physiologic Evaluation of Factors Controlling Glucose Tolerance in Man: Measurement of Insulin Sensitivity and Beta-Cell Glucose Sensitivity from the Response to Intravenous Glucose. J. Clin. Investig..

[42] Khan S.H., Khan A.H., Khan Z.H. (2013). Artificial Pancreas Coupled Vital Signs Monitoring for Improved Patient Safety. Arab. J. Sci. Eng..

[43] Franklin G.F., Powell J.D., Emami-Naeini A. (2002). Feedback Control of Dynamic Systems.

[44] Ogata K. (1997). Modern Control Engineering.

[45] FDA Classify Your Medical Device. http://www.webcitation.org/6ZdUv0YwI.

[46] ROCHE (2008). ACCU-CHEK Spirit Insulin Pump System: Pump User Guide. http://www.webcitation.org/6ZdVHOgBx.

[47] Diabetes.co.uk Basal Bolus—Basal Bolus Injection Regimen. http://www.webcitation.org/6ZdVLD7NE.

[48] Lee E.A., Seshia S.A. (2015). Introduction to Embedded Systems, A Cyber-Physical Systems Approach.

[49] Silva L.C., Perkusich M., Bublitz F.M., Almeida H.O., Perkusich A. A Model-Based Architecture for Testing Medical Cyber-Physical Systems. Proceedings of the 29th Annual ACM Symposium on Applied Computing (SAC).

[50] Ptolemaeus C. (2014). System Design, Modeling, and Simulation Using Ptolemy II.

[51] Silva L.C., Almeida H.O., Perkusich A., Perkusich M. (2015). A Model-Based Approach to Support Validation of Medical Cyber-Physical Systems. http://sites.google.com/site/mbatomcps/.

[52] NIH (2014). Asthma. http://www.nhlbi.nih.gov/health/health-topics/topics/asthma.

[53] WHO (2013). 10 Facts about Diabetes. http://www.webcitation.org/6ZdVT2Scz.

[54] WHO (2012). World Health Statistics. http://www.who.int/gho/publications/world_health_statistics/2012/en/.

[55] Gomes M.B., Lerario A.C. (2009). Gerenciamento Eletrônico do Diabetes. Dir. Soc. Bras. Diabetes.

[56] Kitchenham B., Pickard L., Pfleeger S.L. (1995). Case studies for method and tool evaluation. IEEE Softw.

[57] Washizaki H., Yamamoto H., Fukazawa Y. A metrics suite for measuring reusability of software components. Proceedings of the 2003 Ninth International Software Metrics Symposium.

[58] Mathworks Stateflow, 2014. http://www.mathworks.com/products/stateflow/.

[59] Utting M., Legeard B. (2007). Practical Model-Based Testing: A Tools Approach.

[60] Bacic M. On hardware-in-the-loop simulation. Proceedings of the 44th IEEE Conference on Decision and Control and the European Control Conference (CDC-ECC).

